# Engaging online students by *activating* ecological knowledge

**DOI:** 10.1002/ece3.6739

**Published:** 2020-09-01

**Authors:** Stacy L. Hines, Anthony J. Vedral, Amanda E. Jefferson, J. Marcus Drymon, Mark S. Woodrey, Sarah E. Mabey, Eric L. Sparks

**Affiliations:** ^1^ Department of Wildlife, Fisheries, and Aquaculture Mississippi State University Mississippi State MS USA; ^2^ Coastal Research and Extension Center Mississippi State University Biloxi MS USA; ^3^ Mississippi‐Alabama Sea Grant Consortium Ocean Springs MS USA; ^4^ Grand Bay National Estuarine Research Reserve Moss Point MS USA; ^5^ School of Education, Civic Leadership, and Social Change Hiram College Hiram OH USA

**Keywords:** ecology, education, facilitation, natural resources, online learning

## Abstract

The current COVID‐19 pandemic has forced the global higher education community to rapidly adapt to partially or fully online course offerings. For field‐ or laboratory‐based courses in ecological curricula, this presents unique challenges. Fortunately, a diverse set of active learning techniques exists, and these techniques translate well to online settings. However, limited guidance and resources exist for developing, implementing, and evaluating active learning assignments that fulfill specific objectives of ecology‐focused courses. To address these informational gaps, we (a) identify broad learning objectives across a variety of ecology‐focused courses, (b) provide examples, based on our collective online teaching experience, of active learning activities that are relevant to the identified ecological learning objectives, and (c) provide guidelines for successful implementation of active learning assignments in online courses. Using The Wildlife Society's list of online higher education ecology‐focused courses as a guide, we obtained syllabi from 45 ecology‐focused courses, comprising a total of 321 course‐specific learning objectives. We classified all course‐specific learning objectives into at least one of five categories: (a) Identification, (b) Application of Concepts/Hypotheses/Theories, (c) Management of Natural Resources, (d) Development of Professional Skills, or (e) Evaluation of Concepts/Practices. We then provided two examples of active learning activities for each of the five categories, along with guidance on their implementation in online settings. We suggest that, when based on sound pedagogy, active learning techniques can enhance the online student's experience by *activating* ecological knowledge.

## Graphical abstract

The current COVID‐19 pandemic has forced the global higher education community to rapidly adapt to partially or fully online course offerings. For field‐ or laboratory‐based courses in ecological curricula, this presents unique challenges. Fortunately, a diverse set of active learning techniques exist, and these techniques translate well to online settings. However, limited guidance and resources exist for developing, implementing, and evaluating active learning assignments that fulfill specific objectives of ecology‐focused courses. To address these informational gaps, we (a) identify broad learning objectives across a variety of ecology‐focused courses, (b) provide examples, based on our collective online teaching experience, of active learning activities that are relevant to the identified ecological learning goals, and (c) provide guidelines for successful implementation of active learning assignments in online courses.

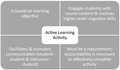

## 1 INTRODUCTION

The current COVID‐19 pandemic has impacted most aspects of daily life, including but not limited to educational instruction. Because of state and federal quarantine orders, colleges and universities around the world have been relegated to providing virtual instruction rather than face‐to‐face education. Traditional face‐to‐face pedagogical approaches (e.g., lecture‐based approach) are likely ineffective in fully engaging students in an online setting (Garrison, 2003; Slavich & Zimbardo, 2012). Thus, to deliver content effectively, instructors must adapt their approaches in accordance with research‐based methods deemed successful for online instruction (Crawford‐Ferre & Wiest, 2012; Schrum, Burbank, Engle, Chambers, & Glassett, 2005). Successful online instruction is best achieved when the instructor assumes the role of a facilitator, thereby guiding the students’ learning experiences (Berge, 1995; Crawford‐Ferre & Wiest, 2012; Slavich & Zimbardo, 2012; Vilppu, Södervik, Postareff, & Murtonen, 2019). This strategy shifts the emphasis of online curriculum development from content‐focused to learning‐focused (Slavich & Zimbardo, 2012; Vilppu et al., 2019).

The purpose of learning‐focused curriculum is to facilitate students’ deep learning process by directing them in activities to help build their knowledge (Postareff & Lindblom‐Ylӓnne, 2008; Trigwell, Prosser, & Waterhouse, 1999). In the online environment, instructors should not merely transmit knowledge through passive learning activities such as reading or watching video lectures, where students learn mainly by receiving information (Dixson, 2010; Vilppu et al., 2019). Rather, in addition to passive learning activities, effective online teaching must include the promotion of active, self‐regulated learning (Vermunt, Vrikki, Warwick, & Mercer, 2017). Instructors should initiate and guide the students’ deep learning processes so they are encouraged to actively construct their own understanding (Vilppu et al., 2019). Active learning is achieved when the students apply the information they have learned (Meyers & Jones, 1993; Slavich & Zimbardo, 2012).

**FIGURE 1:**
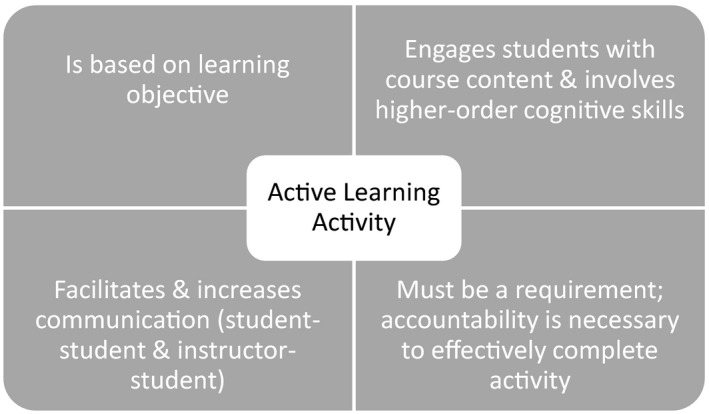
Key components, based on current published literature, of a given active learning activity designed to meritoriously increase student engagement

Student engagement is a primary component of effective teaching. Active learning activities have the potential to increase student engagement in online courses (Chickering & Ehrmann, 1996). Dixson (2010) determined from a survey of 186 higher education students that student engagement was successful when active learning assignments engaged the students with (a) the content, (b) the instructor, and (c) other students. Students’ perceptions of their engagement levels were not dictated by the specific type of active learning activity. Rather, these perceptions were dictated by the students’ sense of connection and increased when multiple opportunities for connection were provided (Dixson, 2010). We identified four key elements for developing and effectively utilizing active learning activities from the literature (Figure 1). First, active learning activities should be centered on the learning objective (Koontz, Li, & Compora, 2006). Second, active learning activities should foster student engagement with content and higher‐order cognitive skills (Meyers & Jones, 1993; Slavich & Zimbardo, 2012; Vermunt et al., 2017). Third, instructors must require students to complete the work because students put forth less effort when they are not held accountable for completing tasks (Dixson, 2010; Janssens, Boes, & Wante, 2002). Fourth, active learning activities must promote communication because students perceive activities as successful when the activities enhance communication among students and/or between students and the instructor (Dixson, 2010).

Ecology‐focused courses are particularly challenging to deliver online with meaningful student engagement. The concepts and applications associated with these courses have traditionally been viewed as very “hands‐on,” classically requiring in‐person instruction to effectively deliver information. Many ecology‐focused courses are taught partly or entirely in the field and laboratory, where instructors provide information on basic concepts and also incorporate unscripted teaching moments. For these reasons, creating online ecology‐focused courses or transitioning conventionally field‐based ecology‐focused courses to an online format can be difficult.

Regardless of course format, the primary role of the online instructor is to ensure the learning objectives are accomplished (Koontz et al., 2006). Therefore, active learning assignments should be developed based on the specific learning objectives for the course. From observational data and experience, ecology‐focused courses seem to possess consistent themes (i.e., learning objectives). However, limited guidance and resources exist for developing, implementing, and evaluating active learning assignments that fulfill specific objectives of ecology‐focused courses. To address these informational gaps, we (a) identify broad learning objectives across a variety of ecology‐focused courses, (b) provide examples, based on our collective online teaching experience, of active learning activities that are relevant to the identified ecological learning objectives, and (c) provide guidelines for successful implementation of active learning assignments in online courses.

## 2 MATERIALS AND METHODS

We used The Wildlife Society's (TWS) list of online higher education ecology‐focused courses as a guide for identifying and classifying our initial categories of learning objectives (The Wildlife Society, 2020). First, we reviewed the fields of study (e.g., categories of ecology‐focused courses) for courses offered online that were listed on the TWS website. Specific fields of study listed included: Biology; Botany; Communications; Ecology; Humanities; Physical Sciences; Policy, Administration, and Law; Quantitative Sciences; Statistics; Sustainability; Wildlife and Natural Resource Management; Wildlife Biology; and Zoology. Collectively, we have taught courses in most of these fields of study; we only lack higher education instructional experience in courses dedicated to the Humanities field of study. After reviewing the fields of study, we used our collective experience to develop a preliminary list of five learning objective categories that we postulated were broad enough to encompass all of the course‐specific learning objectives for the ecology‐focused fields of study.

Next, we gathered information directly through institutional websites and Google searches to verify our initial categorization of course‐specific learning objectives for online courses that aligned with the list on the TWS website. We browsed course catalogs and departmental pages to locate syllabi for courses considered to align with fields similar to those listed on the TWS website. In cases where syllabi were not linked on institutional pages, we used key term Google searches to find available syllabi for courses. Key terms included the name of the institution paired with a field of study and the words “syllabus” and “online.” We only obtained syllabi for courses currently offered at an institute; however, course syllabi were not restricted to the current academic year. Dates listed on procured syllabi ranged from 1999 to 2020. Additionally, some syllabi that we obtained were listed as “example syllabi,” meaning they were from a previous year of the course, but the date was removed. We were not able to obtain syllabi from every institution listed on the TWS website due to limited accessibility. However, we did obtain syllabi from every field of study listed on TWS webpage.

Then, we compared the course‐specific learning objectives to the initial framework for our five learning objective categories. We made slight modifications to two of our preliminary categories to better align them with consistent themes across the procured course‐specific learning objectives. Our final five categories of learning objectives were (a) Identification, (b) Application of Concepts/Hypotheses/Theories, (c) Management of Natural Resources, (d) Development of Professional Skills, and (e) Evaluation of Concepts/Practices.

**FIGURE 2:**
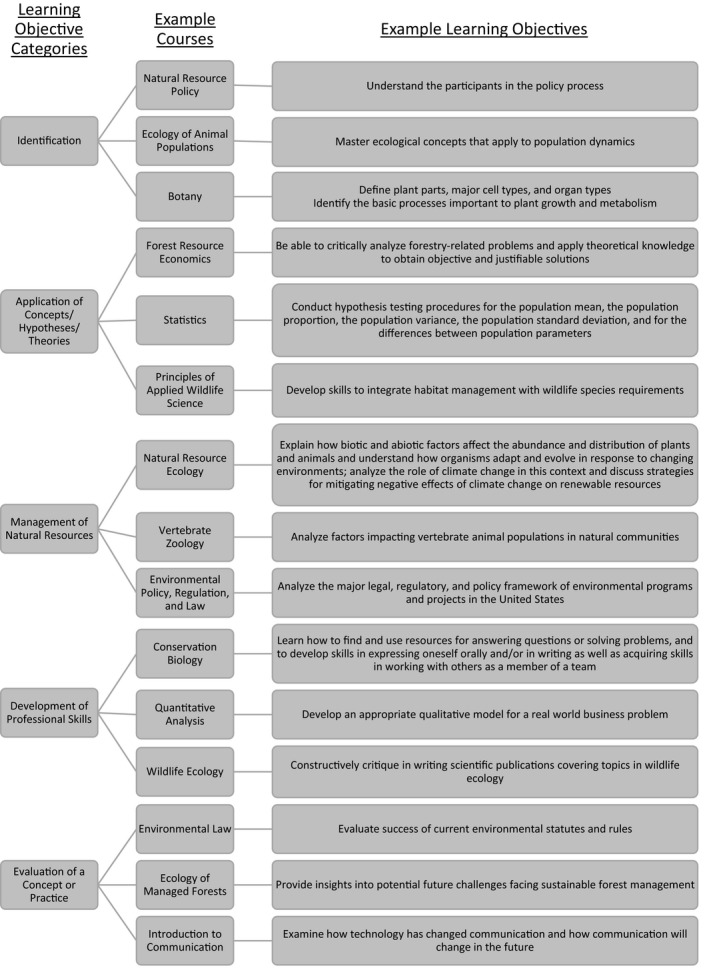
Examples of higher education ecology‐focused courses and course‐specific learning objectives that aligned with our five categories of learning objectives. We obtained these learning objectives from course syllabi procured from institutional pages and Google searches

Finally, we categorized every course‐specific learning objective listed on each syllabus into one of our five learning objective categories. Specifically, one person categorized all of the course‐specific learning objectives based on keyword terms and synonyms of keyword terms we developed for each of our learning objective categories (Table 1). For example, keyword terms for our Identification learning objective category included (a) define, (b) describe, (c) identify, (d) learn, and (e) understand. Many course‐specific learning objectives were broad and encompassed several keywords, thereby matching more than one of our categories. For example, where identification of a term was a learning outcome, the course‐specific learning objective also often included the application of the term; therefore, this particular learning objective would align with our Identification category and our Application of Concepts/Hypotheses/Theories category. In cases like this one, the broadly written course‐specific learning objective was given credit for multiple categories (Table 1; Figure 2; Appendix A). It should be noted that the intent of this categorization scheme is not to rank the quality of courses or make an assertion that a course is lacking in certain aspects. The sole purpose of the exercise was to determine whether our five learning objective categories indeed explained most course‐specific learning objectives that were listed on syllabi across many ecology‐focused courses.

**TABLE 1:** Methodology for assigning learning objectives of ecology‐focused courses into our five author‐defined learning objective categories

Learning objective category	Keyword terms	Categorization of course objectives
Identification	Define, describe, identify, learn, understand	In the identification category, we included learning objectives related to the identification of terminology and species. The identification category was the most inclusive category of all our learning objective categories. Many course objectives were broad and encompassed several keywords that matched more than one of our categories. For example, where identification of terminology was a learning outcome, the learning objective often included the application of the term (e.g., to manage natural resources). In those cases, the objective was given credit for multiple categories
Application of concepts/hypotheses/theories	Apply, develop, formulate, predict, provide, suggest	In this category, we looked for indication that a knowledge base was applied to complete a task or creation of a plan or project; higher‐order thinking tasks were included in this category. Many learning objectives were broad and encompassed several keywords that matched more than one of our categories. For example, many learning objectives that included analyzing, such as applying theoretical knowledge to analyze a problem, also included the keyword term “evaluate.” In those cases, the objective was given credit for multiple categories
Management of natural resources	Conservation, laws, management, natural resources, policy, populations, regulations, sustainability	If a learning objective was related to conservation of natural resources or wildlife, it qualified for this category. Many of the learning objectives inherently featured a process of identification and therefore qualified for the identification category as well. In many cases, such as assessing the role of collaborative efforts in wildlife management, the learning objective also qualified for application or evaluation categories dependent upon context
Development of professional skills	Career paths, case studies, communication, laboratory skills, models, scientific method, real‐world problems, technological skills	Objectives that focused on professional practice or understanding of a skill qualified for this category. Some learning objectives (e.g., practicing professional communication skills through a discussion related to conservation practices) also qualified for additional categories
Evaluation of concepts/practices	Analyze, argue, assess, critique, determine, effects, evaluate, review	In order to qualify for this category, learning objectives had to involve the use of critical thinking skills to evaluate a concept or a common professional practice. Objectives that qualified for this category typically involved higher‐order thinking where the application of a process, management, or change was evaluated. In many cases, learning objectives in this category (e.g., the evaluation of applying an environmental management policy) also qualified for other categories

## 3 RESULTS

### 3.1 Learning objective categories

We obtained syllabi from 45 ecology‐focused courses (Table 1; Appendix A). Each course syllabus had 3–18 learning objectives. We reviewed a total of 321 course‐specific learning objectives. All course‐specific learning objectives were classified into at least one of our following five ecology‐focused learning objective categories: (a) Identification, (b) Application of Concepts/Hypotheses/Theories, (c) Management of Natural Resources, (d) Development of Professional Skills, or (e) Evaluation of Concepts/Practices.

Learning objectives categorized as Identification were included in 100% of courses (45 out of *n* = 45) and were mostly related to the identification of species (i.e., plants, animals), anatomy, types of data and graphical representations, terminology and definitions, laws, and ecological processes (Table 1; Figure 2; Appendix A). For example, a learning objective from a botany course that aligned with our Identification category was, “Define plant parts, major cell types, and organ types. Identify the basic processes important to plant growth and metabolism” (Michot; n.d.; Botany; American Public University System).

Learning objectives categorized as Application of Concepts/Hypotheses/Theories were included in 91% of identified courses (41 out of *n* = 45) and were mostly related to the application of knowledge to ecological concepts/principles, natural selection, global distribution of biotic components (e.g., biomes, plant communities, animals), universal laws (e.g., thermodynamics, conservation of mass), the scientific method/research, policies/laws, biological hierarchy, and evolution (Table 1; Figure 2; Appendix A). For example, a learning objective from a forestry course that aligned with this category was, “Students should be able to critically analyze forestry‐related problems and apply theoretical knowledge to obtain objective and justifiable solutions" (Grala; n.d.; Forest Resource Economics; Mississippi State University).

Learning objectives categorized as Management of Natural Resources were included in 71% of courses (32 out of *n* = 45) and were mostly related to biotic populations (e.g., plants, animals), abiotic components (e.g., water, nutrients), interdependency of abiotic and biotic components, conservation and policies that support conservation, and impacts of humans and other disturbance activities (Table 1; Figure 2; Appendix A). For example, a learning objective from an ecology course that aligned with this category was, “Explain how biotic and abiotic factors affect the abundance and distribution of plants and animals and understand how organisms adapt and evolve in response to changing environments; analyze the role of climate change in this context and discuss strategies for mitigating negative effects of climate change on renewable resources” (Johnson, 2014; Natural Resource Ecology; University of Florida).

Learning objectives categorized as Development of Professional Skills were included in 80% of courses (36 out of *n* = 45) and were mostly related to population/habitat management, applying research and statistical analyses to conservation and management, improving oral and written communication, applying laws and policies to conservation and management, and obtaining/comprehending scientific literature (Table 1; Figure 2; Appendix A). For example, a learning objective from a conservation biology course that aligned with this category was, “Students will learn how to find and use resources for answering questions or solving problems, and to develop skills in expressing oneself orally and/or in writing as well as acquiring skills in working with others as a member of a team” (Chynoweth, 2018; Conservation Biology; Utah State University).

Learning objectives categorized as Evaluation of Concepts/Practices were included in 64% of courses (29 out of *n* = 45) and were mostly related to the evaluation of policies/laws, abiotic and biotic processes/cycles, scientific method and inquiry, technological advances, impacts to conservation and management, written documents (e.g., laws, scientific literature), evolution, conservation, and human impacts to systems (Table 1; Figure 2; Appendix A). For example, a learning objective from an environmental policy and law course that aligned with this category was, “Evaluate success of current environmental statutes and rules” (Brekken; 2005; Environmental Law; Oregon State University).

### 3.2 Example active learning activities for online courses

We provide two examples of active learning activities for each of our five ecology‐focused learning objective categories. Most of these examples have been used for instruction in online and/or face‐to‐face ecology‐related courses. For each activity, we give a brief summary including (a) the course title, education level, and purpose of the activity; (b) a summary of the activity and steps for completion; and (c) potential modifications for other ecology‐focused courses. Detailed descriptions of the activities and tips for converting the activities for online delivery are provided in Appendix B. While active learning activities have the potential to increase student engagement, we have not specifically compared the effectiveness of these activities with that of traditional lecture approaches. However, each of our example active learning activities addresses the four key elements for developing and effectively utilizing active learning activities that we identified from the literature (Figure 1).

#### 3.2.1 Identification

*Example 1—Defining characteristics and image boards (* *Appendix* B.1 *):* The defining characteristics and image boards activity has been used in a Wildlife Plant Identification laboratory course at a split upper undergraduate/graduate level. This activity was developed as a prelaboratory activity to aid students in identification of plants they would collect in the field. Students researched and developed a written description of plant parts (e.g., flower, leaves) that helped categorize the plant species into its group (i.e., taxonomic group such as family or genus, growth habit group such as graminoid or forb). Next, students conducted an online image search to find several images that aligned with the written description. Students applied this knowledge in the field to help them identify the correct type of plant to investigate the identity of using plant identification applications (e.g., iNaturalist). This activity could be modified for any course (e.g., introductory science courses, ornithology, botany) where the learning objective is to identify/group objects by using visually describable defining characteristics.

*Example 2—Diversity and taxonomic rankings (* *Appendix* B.2 *):* The diversity and taxonomic rankings activity has been used in a Shark and Ray Biology field course at the upper undergraduate level. This activity was developed to highlight the wide diversity of sharks in the northern Gulf of Mexico while providing students with practice using a dichotomous key. First, students were presented with a Google Slides file that contained instructions and photographs of preserved shark and ray specimens. Then, students were given two hours to identify 34 specimens to a predetermined taxonomic ranking: order, family, or species. During the two‐hour period, the professor answered clarifying questions about dichotomous key terminology or difficult‐to‐identify specimens using the chat function in Zoom. Finally, at the end of the two‐hour period, correct answers were shared and discussed with the class. This activity could be modified for any course (e.g., vertebrate zoology or other taxon‐specific courses) where the objective is to introduce students to diverse new taxa while familiarizing them with the intricacies of a dichotomous key.

#### 3.2.2 Application of concepts/hypotheses/theories

*Example 1—Biological hierarchy (* *Appendix* B.3 *):* The biological hierarchy activity has been used in a biology course at the introductory undergraduate level. This activity was developed as a lecture summary activity to aid students in applying the concepts and terminology associated with the hierarchy of biological organization (e.g., biosphere, ecosystems, communities). Students applied knowledge by developing their own terminology definitions and study aids. Next, students applied definitions of hierarchy by developing a hierarchical relationship of “everyday” objects (e.g., balls, writing utensils). Finally, students applied definitions of terms to classify levels within the biological hierarchy through critical thinking applications and drawing activities. This activity could be modified for any course (e.g., introductory science courses, botany, ecology) where the learning objective is to apply knowledge of a hierarchical classification system.

*Example 2—Coastal restoration plan (* *Appendix* B.4 *):* The coastal restoration plan activity has been used in an applied Coastal Restoration course at a split upper undergraduate/graduate level. This two‐part activity (a) was developed to help students critically think through the full process for restoration project development and immediately apply knowledge gained by creating a tangible product and (b) allowed for self‐assessment of knowledge gained throughout the course by having students revise the product developed on day 1 into a final product turned in at the end of the course. Immediately after reviewing the syllabus on the first day of the course, students are provided with a template and given instructions for drafting a short (<5 pages including figures and tables) restoration plan on a topic/habitat of their choice. The draft must be submitted by the end of the first day. The students conduct their own research to compile the required information; however, they can interact with the instructor and their classmates through discussion board threads. The instructor is available to answer questions until 5 p.m., but only from the perspective of a potential funder, permitting agency, or stakeholder. On the second day of the course, students are informed that they are expected to continue developing this plan over the duration of the course, present their plan to the class, incorporate feedback, and submit the plan for a final grade. This activity could be modified for any course where a learning objective is to apply knowledge for the conservation and/or restoration of natural resources (e.g., wildlife management, fisheries management, natural resource management, conservation).

#### 3.2.3 Management of natural resources

*Example 1—Restoring native prairie plant community (* *Appendix* B.5 *):* The restoring native prairie plant community activity has been used in a Wildlife Plant Identification lecture course at a split upper undergraduate/graduate level. This activity was developed as a lecture summary activity to provide students with skills to evaluate the effectiveness of a real seed mixture used for restoring a native prairie plant community as wildlife habitat. Students were given the common names of plant species as listed on an actual seed mix packet that was used to restore native prairie plant community in the Southeastern United States. Next, students were instructed to research background information (e.g., growth duration, native range, growing conditions) about, and wildlife use of, each plant species. Finally, students were asked to evaluate the effectiveness of this seed mixture and support their assessment with facts gathered from their research. This activity could be modified for any course (e.g., ecology, wildlife management, natural resource management) where the learning objective is to evaluate a tool used to restore a native ecosystem.

*Example 2—Google Earth Mississippi estuaries journey (* *Appendix* B.6 *):* The Google Earth Mississippi estuaries journey activity has been used in high school AP Environmental Science and Honors Marine Biology classes and in informal education through a youth version of Mississippi's Master Naturalist Program called “Student Naturalist.” In this activity, students apply their knowledge of estuarine geography and natural and anthropogenic influences (e.g., nutrient runoff, dredging, development) to answer questions regarding potential impacts (e.g., eutrophication, coastal erosion, habitat degradation, hydrology changes) and make suggestions for mitigation methods. First, students enter GPS coordinates into the Google Earth program; these initial coordinates virtually place them upriver in the Pascagoula River. They are then instructed to move in predetermined directions throughout waterways in the region, stopping at specific areas to try to determine what activities (e.g., golf courses, mining, refineries, roadways) they might see that are impacting that area and adjacent habitat. Lastly, the students are asked to supply potential solutions for reducing the impacts. This activity could be adapted globally to a multitude of environmental, geographic, and anthropological courses where the learning objective is management of natural resources.

#### 3.2.4 Development of professional skills

*Example 1—Initial and reply discussion board posts (* *Appendix* B.7 *):* The initial and reply discussion board posts activity was used in an Environmental Law course at the introductory undergraduate level. This activity was developed as a lecture summary activity to provide students with the opportunity to apply environmental laws to their professional life and practice communication skills. Students were provided Web links to administrative code posted in a state town hall forum as required by public notice executive orders. Next, students were asked to write an initial discussion board post in which they formulated their opinion on three sections of the Administrative Code that were listed: two Administrative Code sections were chosen by the instructor and one was chosen by each student. Two days after the initial post was due, students were required to post a reply to any one classmate's initial post. For the reply post, students were asked to explain if they agreed or disagreed with their classmate and why. This activity could be modified for any course (e.g., introductory or upper undergraduate or graduate levels) where the learning objective is to develop professional opinions and practice communication skills.

*Example 2—Field notebook (* *Appendix* B.8 *):* The field notebook activity has been used at the introductory and upper undergraduate levels. Creating and maintaining a field notebook supports the development of multiple, broadly transferrable professional skills; as such, this is considered a high‐impact learning activity (Farnsworth, Baldwin, & Bezanson, 2014). Students are required to keep detailed field notebooks to record field observations and standardized data in real time using a model format based on exemplary styles (e.g., Herman, 1986; Montgomerie, 2018; Remsen, 1977). Students submit notes periodically during the course to provide the instructor with an opportunity to provide feedback, coaching for improvement, and prompts to direct future observations. This activity could be modified for any course (e.g., geology, wildlife management, marine ecology) where the learning objective is to practice and develop professional skills centered on observation and description, record‐keeping, and contextualizing direct experience.

#### 3.2.5 Evaluation of a concept or practice

*Example 1—Peer evaluation (* *Appendix* B.9 *):* The peer evaluation activity has been developed, but not yet used for instruction. It will be used in a new Aquatic Biodiversity Conservation course at the graduate level. This activity was developed as a lecture series summary and examination review activity to provide students with an opportunity to practice evaluation of peers’ conservation education interpretative displays. Students are provided with resources, examples, and rubrics to develop their own interpretive display on a topic that will be selected by each student, but related to material from the lecture series on freshwater river ecosystems. Next, students are given instructions regarding evaluation. Finally, students will use a faculty‐developed grading rubric to evaluate classmates’ interpretive displays on the merits of creativity, alignment of learning objectives, scientific accuracy, and execution. This activity could be modified for any course (e.g., introductory, advanced undergraduate, graduate) where the learning objective is for students to develop original works and practice their evaluation skills.

*Example 2—Evaluate results of published literature (* *Appendix* B.10 *):* The evaluate results of published literature activity has been used in a Wildlife Plant Identification lecture course at a split upper undergraduate/graduate level. This activity guides students through evaluating the results of published literature to determine whether it supports a theory that has led to a common management practice. First, students are oriented to the learning objective for the activity in the assignment instructions: “Evaluate the results of the study to determine if it supports or does not support Aldo Leopold's theory of using livestock as a wildlife habitat management tool; to set back the seral stage of succession by consuming grasses, thus increase abundance of forbs” (Leopold, 1933). Next, students are asked several key questions to guide them in pulling pertinent facts from the manuscript that provide supporting evidence for their evaluation. Finally, students are asked an open‐ended evaluation question: “Did the researchers of this manuscript find a treatment effect that would support Aldo Leopold's theory of using cattle or livestock as a wildlife habitat management tool to set back the seral stage of succession?” This activity could be modified for any course (e.g., ecology, zoology, wildlife biology) where the learning objective is to evaluate the results of published literature to determine whether it supports a concept or practice.

## 4 DISCUSSION

The past half century has been marked by a gradual shift in higher education pedagogy from the transmittal model (i.e., “sage on the stage”) to the transformational model (i.e., “guide on the side”) (King, 1993; Slavich & Zimbardo, 2012). This transition was accentuated by the current COVID‐19 pandemic, which has forced the global higher education community to rapidly adapt to partially or fully online course offerings (Crawford et al., 2020). The abrupt switch to emergency remote instruction has required instructors to embrace the role of a facilitator, but instructors lack resources to engage students in online settings. This can be especially difficult for instructors who desire to teach using an active learning pedagogy. For example, many instructors of face‐to‐face and field‐based ecology‐focused courses are prepared to lead classroom lectures, but rely heavily on opportunistic, unscripted outdoor experiences and interactions with students to supplement the classroom material. Fortunately, some in‐person active learning activities can be modified for online instruction. Here, we further the knowledge from the transformational teaching literature (Slavich & Zimbardo, 2012) by providing observations and suggestions for online active learning activities based on our collective use of active learning activities in ecology‐focused courses.

First, online active learning activities designed as lecture summary activities should be relatively short and focused explicitly on the learning objectives. This compels instructors to focus specifically on the primary learning objectives for the course or lesson, rather than on tangential or supplementary topics. As an added benefit, a small number of brief, focused activities provides students with a greater proportion of time to self‐learn the lecture material (i.e., at a comfortable pace and using individualized techniques) and develop their own interpretations (Vilppu et al., 2019). We recommend that (a) fill‐in‐the‐blank activities should be no more than 2–3 pages in length, (b) activities regarding a reading assignment should contain a maximum of 10 questions, and (c) discussion or forum activities should comprise 2–3 main questions.

Second, mandatory submission for active learning assignments can aid online instructors in tracking student attendance. This is particularly important for institutes that rely on federal aid funds. According to the Federal Student Aid handbook, students are considered “in attendance” in an online course when they (a) submit assignments or examinations, (b) post comments in an online discussion, or (c) participate in an interactive tutorial (E‐Campus Solutions Center, 2020; Office of Distance Education & eLearning, 2017). Importantly, mandatory submission of active learning activities does not necessitate evaluation of every assignment by the instructor. Self‐evaluation by the students is considered to be a valuable learning tool; in fact, evaluation is classified as higher‐order learning (Berge, 1995). We evaluated online and face‐to‐face active learning activities using the same strategy; we assigned participation points for fully completed activities and then allowed the students to self‐correct their answers. For example, in an undergraduate Wildlife Plant Identification course, we assigned active learning activities for every weekly lecture; if a student earned 100% participation points for fully completing an activity, then the student was granted access to the answer key for the activity. Although self‐evaluation lessens the required amount of involvement from the online instructor, we still recommend that the instructor provide feedback on every submission to increase student engagement through student–instructor communication (Dixson, 2010; Slavich & Zimbardo, 2012).

Third, we recommend incorporating active learning activities that allow students to guide their own unique instructional journeys. For instance, in our example *Coastal restoration plan* (Appendix B.4), students are asked to design a restoration project with the freedom to choose their own focus area and path to success without much initial guidance. This strategy, known as student‐centered learning, enables students to independently discover the resources available to them. Although student‐driven resource exploration requires the online instructor to spend more time guiding students (Gabriel & Kaufield, 2008; Schrum et al., 2005), it increases student engagement by creating communication opportunities among students and between students and the instructor (Slavich & Zimbardo, 2012). Student‐centered learning also promotes student choice by allowing students to apply course material to their own interests. This further heightens student engagement because each student has the opportunity to play an active part in shaping the course content (Slavich & Zimbardo, 2012).

Fourth, if the online instructor opts to use active learning activities that increase student engagement through student‐to‐student communication, then the instructor must facilitate that communication. Prior to the start of the activity, it is imperative that the online instructor clearly state his or her expectations for courtesy and professional language. The instructor should also designate precise deadlines for student communications. In our experience, most online students wait until the last possible moment (i.e., the deadline) to submit comments and hand in work, which may result in insufficient time to finish a final product. A series of deadlines throughout the activity allows students to complete tasks in a step‐by‐step manner and helps to provide sufficient time for completing the final product and achieving the final learning objective. Additionally, if the online instructor is requiring group work or peer evaluation at any point then he or she should divide the students into groups and set guidelines for the students to follow, thereby fostering a positive virtual environment.

Finally, to create a hospitable learning environment for all students, it is important to integrate classroom equity into activities, especially when considering that certain disadvantages may be amplified by the transition to online courses. Some students may not have the necessary technology or bandwidth to fully partake in some aspects of activities. Therefore, we recommend providing supplemental materials that will allow an alternate path to success. For example, in the *Defining characteristics and image boards* activity (Appendix B.1), an alternative for using a plant identification application on a portable device would be to provide a digital file (e.g., a handout) of a regional field guide of plants. Additionally, students have different levels of proficiency with technology. Therefore, tutorials and/or supplemental manuals should be provided to ensure that lack of familiarity with a computer application does not inhibit learning. Lastly, online learning should embrace the input of different cultures and life experiences. For example, in the *Google Earth Mississippi estuaries journey* activity (Appendix B.6) impacts and mitigation strategies voiced by students might differ depending on socioeconomic status. The inclusion of such aspects into a discussion not only creates an inviting atmosphere for students of different backgrounds, but also benefits the entire class by broadening the perspectives of all the students.

Ecology‐focused courses, especially field‐ and laboratory‐based courses, present a unique challenge for online delivery. In field and laboratory settings, students are granted ample time for discovery, problem‐solving, and reflection, all while receiving concurrent encouragement and guidance from the instructor, who naturally acts as a facilitator. While these in‐person experiences can never be completely replicated in online settings, active learning activities have the potential to increase student engagement in these settings. Research has indicated that active learning activities increased student performance over traditional lecturing methods (Freeman et al., 2014). However, we have not explicitly compared the efficacy of these approaches to traditional (e.g., lecture‐based) pedagogy in an online environment. Future studies could benefit from controlled comparisons between online versions of traditional versus active learning activities to determine specifically how an online student's experience is enhanced by *activating* ecological knowledge.

## CONFLICT OF INTEREST

None declared.

## AUTHOR CONTRIBUTIONS

**Stacy L. Hines:** Conceptualization (lead); investigation (lead); writing – original draft (equal); writing – review and editing (equal). **Anthony J. Vedral:** Investigation (supporting); writing – original draft (equal); writing – review and editing (equal). **Amanda E. Jefferson:** writing – original draft (equal); writing – review and editing (lead). **J. Marcus Drymon:** Conceptualization (supporting); funding acquisition (equal); investigation (supporting); writing – original draft (equal); writing – review and editing (equal). **Mark S. Woodrey:** Conceptualization (supporting); funding acquisition (equal); investigation (supporting); writing – original draft (equal); writing – review and editing (equal). **Sarah E. Mabey:** Conceptualization (supporting); investigation (supporting); writing – original draft (equal); writing – review and editing (equal). **Eric L. Sparks:** Conceptualization (supporting); funding acquisition (equal); investigation (supporting); writing – original draft (equal); writing – review and editing (equal).

## Supporting information

Appendix S1

## Data Availability

All data, products, and examples associated with this publication are fully presented within the text and found on Dryad (https://doi.org/10.5061/dryad.b8gtht79q).

## References

[ece36739-bib-0001] Berge, Z. L. (1995). The role of the online instructor/facilitator. Educational Technology, 35(1), 22–30.

[ece36739-bib-0002] Brekken, C. (2005). Environmental law [syllabus]. Corvallis, OR: College of Agricultural Sciences, Oregon State University Retrieved from http://services.ecampus.oregonstate.edu/syllabi/downloadsyllabus.aspx?docid=2352&file=syllabus.pdf

[ece36739-bib-0003] Chickering, A. W. , & Ehrmann, S. C. (1996). Implementing the seven principles: Technology as a lever. Winona, Minnesota: American Association for Higher Education, Seven Principles Resource Center, Winona State University Retrieved June, 15 2020 from http://sphweb.bumc.bu.edu/otlt/teachingLibrary/Technology/seven_principles.pdf

[ece36739-bib-0004] Chynoweth, M. (2018). Conservation biology [syllabus]. Logan, UT: Department of Wildland Resources, Utah State University Retrieved from https://qcnr.usu.edu/wild/courses/syllabi/WILD%204600%20-%20Mark%20Chynoweth.pdf

[ece36739-bib-0005] Crawford, J. , Butler‐Henderson, K. , Rudolph, J. , Malkawi, B. , Glowatz, M. , Burton, R. , … Lam, S. (2020). COVID‐19: 20 countries' higher education intra‐period digital pedagogy responses. Journal of Applied Learning & Teaching, 3(1), 1–20.

[ece36739-bib-0006] Crawford‐Ferre, H. G. , & Wiest, L. R. (2012). Effective online instruction in higher education. The Quarterly Review of Distance Education, 13(1), 11–14.

[ece36739-bib-0007] Dixson, M. D. (2010). Creating effective student engagement in online courses: What do students find engaging? Journal of the Scholarship of Teaching and Learning, 10(2), 1–13.

[ece36739-bib-0008] E‐Campus Solutions Center (2020). How‐to: Document attendance during remote delivery. How‐to guides. Retrieved from https://www.miamioh.edu/regionals/academics/elearning/ecampus-faculty-staff/eccoe-news/2020/03/attendance-remote-delivery.html

[ece36739-bib-0009] Farnsworth, J. S. , Baldwin, L. , & Bezanson, M. (2014). An invitation for engagement: Assigning and assessing field notes to promote deeper levels of observation. Journal of Natural History Education & Experience, 8, 12–20.

[ece36739-bib-0010] Freeman, S. , Eddy, S. L. , McDonough, M. , Smith, M. K. , Okoroafor, N. , Jordt, H. , & Wenderoth, M. P. (2014). Active learning increases student performance in science, engineering, and mathematics. Proceedings of the National Academy of Sciences of the United States of America, 111(23), 8410–8415. 10.1073/pnas.1319030111 24821756 PMC4060654

[ece36739-bib-0011] Gabriel, M. A. , & Kaufield, K. J. (2008). Reciprocal mentorship: An effective support for online instructors. Mentoring and Tutoring: Partnership in Learning, 16(3), 311–327. 10.1080/13611260802233480

[ece36739-bib-0012] Garrison, D. R. (2003). Cognitive presence for effective asynchronous online learning: The role of reflective inquiry, self‐direction and metacognition. Elements of Quality Online Education: Practice and Direction, 4(1), 47–58.

[ece36739-bib-0013] Grala, R. (n.d.). Forest resource economics [syllabus]. Mississippi State, MS: College of Forest Resources, Mississippi State University Retrieved from https://online.msstate.edu/pdf/forestry/syllabi/FO-6113.pdf

[ece36739-bib-0014] Herman, S. G. (1986). The naturalist's field journal: A manual of instruction based on a system established by Joseph Grinnell. Vermillion, SD: Buteo Books.

[ece36739-bib-0015] Janssens, S. , Boes, W. , & Wante, D. (2002). Portfolio: Een instrument voor toetsing en begeleiding In Dochy F., Heylen L., & van de Mosselaer H. (Eds.), Assessment in onderwijs (pp. 203–224). Utrecht, the Netherlands: LEMMA.

[ece36739-bib-0016] Johnson, S. (2014). Natural resource ecology [syllabus]. Gainesville, FL: Department of Wildlife Ecology and Conservation, University of Florida Retrieved from https://ufwildlife.ifas.ufl.edu/pdfs/Syllabus_NREcology.pdf

[ece36739-bib-0017] King, A. (1993). From sage on the stage to guide on the side. College Teaching, 41(1), 30–35. 10.1080/87567555.1993.9926781

[ece36739-bib-0018] Koontz, F. R. , Li, H. , & Compora, D. P. (2006). Designing effective online instruction: A handbook for web‐based courses. Lanham, MD: Rowman & Littlefield Education.

[ece36739-bib-0019] Leopold, A. (1933). Game management. Madison, WI: University of Wisconsin Press.

[ece36739-bib-0020] Meyers, C. , & Jones, T. B. (1993). Promoting active learning strategies for the college classroom. San Francisco, CA: Jossey‐Bass Inc.

[ece36739-bib-0021] Michot, S. (n.d). Botany [syllabus]. Charles Town, WV: Science Department, American Public University System Retrieved from https://www.apus.edu/z/course-syllabus/SCIN314.pdf

[ece36739-bib-0022] Montgomerie, B. (2018, August 21). Joe Grinnell's Notes [Web log post]. Retrieved from https://americanornithology.org/joe-grinnells-notes/

[ece36739-bib-0023] Office of Distance Education and eLearning (2017). Attendance in online courses Course design and pedagogy. Retrieved from https://resourcecenter.odee.osu.edu/course-design-and-pedagogy/attendance-online-courses

[ece36739-bib-0024] Postareff, L. , & Lindblom‐Ylӓnne, S. (2008). Variation in teachers' descriptions of teaching: Broadening the understanding of teaching in higher education. Learning and Instructing, 18, 109–120. 10.1016/j.learninstruc.2007.01.008

[ece36739-bib-0025] Remsen, J. V. Jr (1977). On taking field notes. American Birds, 31, 946–953.

[ece36739-bib-0026] Schrum, L. , Burbank, M. D. , Engle, J. , Chambers, J. A. , & Glassett, K. F. (2005). Post‐secondary educators' professional development: Investigation of an online approach to enhancing teaching and learning. Internet and Higher Education, 8, 279–289. 10.1016/j.iheduc.2005.08.001

[ece36739-bib-0027] Slavich, G. M. , & Zimbardo, P. G. (2012). Transformational teaching: Theoretical underpinnings, basic principles, and core methods. Educational Psychology Review, 24, 569–608. 10.1007/s10648-012-9199-6 23162369 PMC3498956

[ece36739-bib-0028] The Wildlife Society (2020). Online courses. Retrieved from https://wildlife.org/next-generation/career-development/online-courses/

[ece36739-bib-0029] Trigwell, K. , Prosser, M. , & Waterhouse, F. (1999). Relations between teachers' approaches to teaching and students' approaches to learning. Higher Education, 37(1), 57–70.

[ece36739-bib-0030] Vermunt, J. D. , Vrikki, M. , Warwick, P. , & Mercer, N. (2017). Connecting teacher identity formation to patterns in teacher learning In Clandinin D., & Husu J. (Eds.), The Sage Handbook of research on teacher education (pp. 143–159). Los Angeles, CA: SAGE Publications Ltd.

[ece36739-bib-0031] Vilppu, H. , Södervik, I. , Postareff, L. , & Murtonen, M. (2019). The effect of short online pedagogical training on university teachers' interpretation of teaching–learning situations. Instructional Science, 47, 679–709.

