# Supplementary material for: Engaging online students by activating ecological knowledge
Source: Ecol Evol. 2020 Sep 1;10(22):12472–81. doi: 10.1002/ece3.6739 (PMC7679540; doi:10.1002/ece3.6739)
Supplement: Supplementary file 1 — Appendix S1 [file ECE3-10-12472-s001.docx]

# Appendix S1

# Appendix A. Review of learning objectives from ecology-focused course syllabi obtained from institutional pages and Google searches. The focus of the syllabus searches stems from a list of fields of study for online course offerings posted on The Wildlife Society’s webpage (2020). The “x” indicates that one of the course-specific learning objectives listed on the course syllabi aligned with the learning objective category. Courses with identical titles are listed individually.

| **Course** | **Identification** | **Application of Concepts/ Hypotheses/ Theories** | **Management of Natural Resources** | **Development of Professional Skills** | **Evaluation of Concepts/ Practices** |
| --- | --- | --- | --- | --- | --- |
| **Aquatic Animal Conservation Issues** | x | x | x | x | x |
| **Biology** | x | x | x | x | x |
| **Botany** | x | x |  |  | x |
| **Botany** | x |  | x | x |  |
| **Chemistry** | x | x |  | x | x |
| **Communication and Practice of Science** | x | x |  | x |  |
| **Communications and Research** | x | x |  | x | x |
| **Community Ecology** | x | x | x | x |  |
| **Conservation Biology** | x | x | x |  | x |
| **Conservation Biology** | x | x | x | x | x |
| **Ecology** | x | x | x | x | x |
| **Ecology** | x | x | x |  |  |
| **Ecology of Animal Populations** | x | x | x | x |  |
| **Ecology of Managed Forests** | x |  | x | x | x |
| **Ecology of Our Changing World** | x | x |  | x |  |
| **Emerging Issues in Forest Ecosystems** | x | x | x | x |  |
| **Environmental Law** | x | x | x | x | x |
| **Environmental Policy, Regulation, and Law** | x | x | x | x | x |
| **Forest Resource Economics** | x | x | x | x | x |
| **Integrated Principles of Biology I** | x | x | x | x | x |
| **Introduction to Chemistry with Lab** | x | x |  | x | x |
| **Introduction to Communication** | x | x |  | x | x |
| **Introduction to the Marine Environment** | x | x | x |  | x |
| **Introduction to Wildlife Management** | x | x | x |  | x |
| **Managing the Wilderness Resource** | x | x | x | x | x |
| **Monitoring and Assessment in Natural Resource and Environmental Management** | x | x | x | x |  |
| **Natural Resource Ecology** | x | x | x | x | x |
| **Natural Resource Policy** | x |  | x | x |  |
| **Physics I with Lab** | x | x |  |  |  |
| **Practical Legal Concepts for Natural Resource Professionals** | x | x | x | x | x |
| **Preserving Nature** | x | x | x | x |  |
| **Principles and Practices of Applied Wildlife Science** | x | x | x | x | x |
| **Principles of Statistics** | x | x |  | x |  |
| **Quantitative Analysis** | x | x |  | x | x |
| **Quantitative Analysis** | x | x |  | x |  |
| **Soil Science** | x | x | x | x | x |
| **Statistics** | x | x |  | x | x |
| **Statistics** | x | x |  | x |  |
| **The Surface of the Earth** | x | x | x | x |  |
| **Vertebrate Zoology** | x | x | x |  |  |
| **Wetlands** | x |  | x |  | x |
| **Wilderness in the American Context** | x | x | x |  | x |
| **Wilderness Management Planning** | x | x | x | x | x |
| **Wildlife Ecology** | x | x | x | x | x |
| **Wildlife Management** | x | x | x | x | x |
| **Total** | **45** | **41** | **32** | **36** | **29** |

**Appendix B.** Active learning activity examples and resources. Following is a table of contents for Appendix B that lists two sets of active learning activity examples, authors, and email addresses for each of the learning objective categories identified as most relevant to ecology-focused courses.

|  | **Active learning activity examples** | **Authors** | **Email addresses** |
| --- | --- | --- | --- |
| **Identification** | | | |
| B.1 | Defining characteristics and image boards | Stacy L. Hines | sla335@msstate.edu |
| B.2 | Diversity and taxonomic rankings | J. Marcus Drymon | marcus.drymon@mssstate.edu |
| **Application of Concepts/Hypotheses/Theories** | | | |
| B.3 | Biological hierarchy | Stacy L. Hines | sla335@msstate.edu |
| B.4 | Coastal restoration plan | Eric L. Sparks | eric.sparks@msstate.edu |
| **Management of Natural Resources** | | | |
| B.5 | Restoring native prairie plant community | Stacy L. Hines | sla335@msstate.edu |
| B.6 | Google Earth Mississippi estuaries journey | Anthony J. Vedral | anthony.vedral@msstate.edu |
| **Development of Professional Skills** | | | |
| B.7 | Initial and reply discussion board posts | Stacy L. Hines | sla335@msstate.edu |
| B.8 | Field notebook | Sarah E. Mabey &  Mark S. Woodrey | mabeyse@hiram.edu  msw103@msstate.edu |
| **Evaluation of Concepts/Practices** | | | |
| B.9 | Peer evaluation | Stacy L. Hines | sla335@msstate.edu |
| B.10 | Evaluate results of published literature | Stacy L. Hines | sla335@msstate.edu |

**Appendix B.1.** Defining characteristics and image boards

**Learning objective category:** Identification

**Course & Course Level:** Wildlife Plant Identification laboratory course at a split upper undergraduate/graduate level. This activity could be modified for any course (e.g., introductory science courses, ornithology, botany) where the learning objective is to identify/group objects by using visually describable defining characteristics.

**Class Type:** Lecture/Lab; Lab; Online

**Class Size:** 1–25

**Intended Audience:** Ecology-Related Sciences Major; Non-Traditional Student; 2-year College; 4-year College; University

**Required Learning Time:** 30-minutes to one-hour portion of a 3-4-hour lab period

**Prerequisite Student Knowledge:** Students should be familiar with plant anatomy and common anatomical terms, plant taxonomy and basic taxonomic levels, and how to conduct Google image searches.

**Prerequisite Teacher Knowledge:** Instructors should be familiar with plant anatomy and taxonomy, common plant taxonomic groups and their defining characteristics in the student-population targeted region (i.e., Southeastern USA, state of North Carolina, or city of Starkville, MS), web-based resources to research defining characteristics of groups, and how to conduct Google image searches.

**Description:** This activity was developed as a pre-lab activity to aid students in identification of plants they would collect in the field. Students researched and developed a written description of plant parts (i.e., flower, leaves) that helped categorize the plant species into its group (i.e., taxonomic group such as Family or Genus, growth habit group such as graminioid or forb). Next, students conducted an online image search to find several images that aligned with the written description. Students applied this knowledge in the field to help them identify the correct type of plant to investigate the identity of using plant identification applications (i.e., iNaturalist).

**Assessment Type:** Assignment (completed prior to lab field work)

**Activity Details**

Complete the pre-lab work to help you with field identification of plant species within specific plant groups. The plant group may be growth habits (e.g., forb, graminoid, vine, shrub, or tree) or taxonomic classification (e.g., family or genus).

First, conduct research to determine the description of the defining characteristic of the plant parts given for each specific plant group. For example, the plant parts given may be the flower or leaves. The **defining characteristic** should be described in enough detail so a mental image of the plant part is created. Reliable online resources to help you describe the defining characteristics include Encyclopedia Britannica ([https://www.britannica.com/](about:blank)), higher education or extension publications (URLs that end in ‘.edu’), government publications (URLs that end in ‘.gov’), or USDA Plants Database ([https://plants.sc.egov.usda.gov/java/](about:blank)).

Second, conduct a Google image search of the plant group and select several (more than 3) images that visually represent the entire plant and the defining characteristics. If any examples of plants (genera or species) are provided, then also obtain images of the examples. When examples are provided, the plants are found at minimum, throughout most of the Southeastern USA, but most plants are located throughout the USA/Canada.

For example: Plant group is genus *Plantago*, defining characteristics are leaves and flowers, an example is Virginia Plantain (*Plantago virginica*; Figure B.1).

Resource for genus *Plantago* defining characteristics: *Plantago* plant genus:

Encyclopedia Britannica. Retrieved 18 June 2020 from [https://www.britannica.com/plant/Plantago](about:blank)

Resource for Plant Taxonomy: Search for “*Plantago*” on USDA Plants Database [https://plants.sc.egov.usda.gov/java/](about:blank)

All images were copied and pasted from the following Google image searches; “*Plantago*” and “*Plantago virginica*”


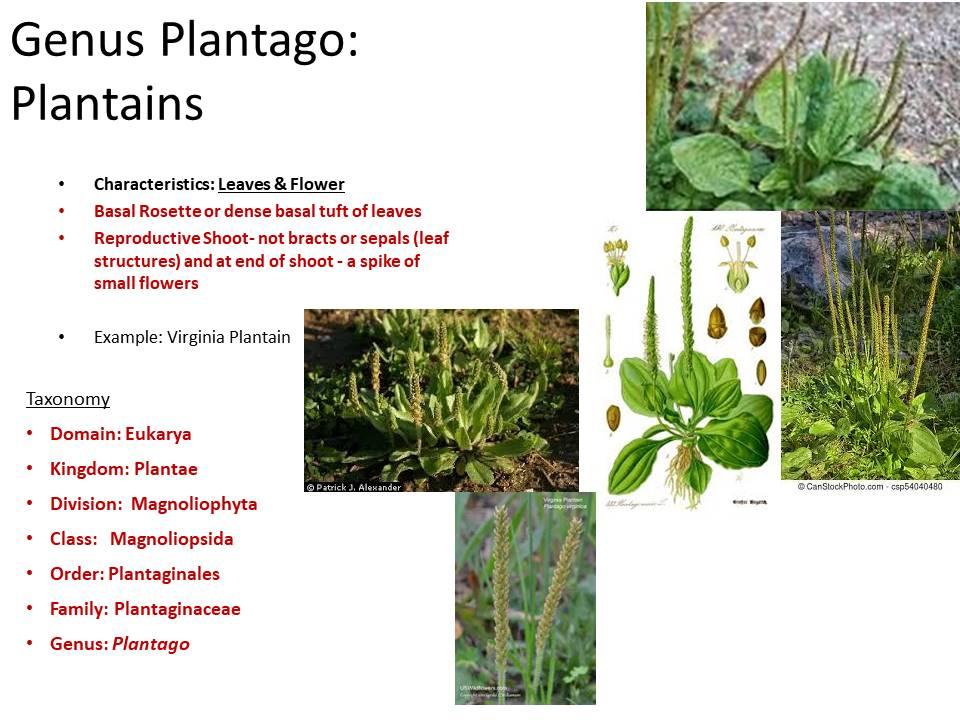


Figure B.1. Example defining characteristics and image board for the genus *Plantago*.

**Tips for executing this activity successfully online:** First, provide explicit directions and clearly define any unique terms. For example, I declared the minimal number of images for an image board and defined the term “defining characteristics.” Second, provide guidance to the students by listing the plant parts for students to research and a list of reliable resources and URLs for the students to use to conduct the research. Third, provide an example of the completed activity so students will have a clear understanding of your expectations. In the Wildlife Plant Identification course, I provided a video tutorial of how to complete the defining characteristics and image board activity. In the video, I provided step-by-step instructions as outlined above. I recommend providing both an instructional video and the written instructions.

**Appendix B.2.** Diversity and taxonomic rankings

**Learning Objective Category:** Identification

**Course & Course Level:** Shark and Ray Biology field course at an undergraduate level. This activity could be modified for any course (e.g., vertebrate zoology or other taxon-specific courses) where the objective is to introduce students to diverse new taxa while familiarizing them with the intricacies of a dichotomous key

**Class Type:** Lecture/Lab; Lab; Online

**Intended Audience:** Biology-Related Sciences Major; Non-Traditional Student; 2-year College; 4-year College; University

**Required Learning Time:** 3 hours of a 3-4-hour lab period

**Prerequisite Student Knowledge:** Students should be familiar with shark anatomy and basic taxonomic levels. Students must also be familiar with a dichotomous key.

**Prerequisite Teacher Knowledge:** Instructors should be familiar with shark anatomy and taxonomy, common shark species and their defining characteristics in the student-population targeted region (i.e., northern Gulf of Mexico, south Atlantic Bight), and the particular dichotomous key being used.

**Description:** This activity was developed to highlight the wide diversity of sharks in the northern Gulf of Mexico while providing students with practice using a dichotomous key. First, students were presented with a Google Slides file that contained instructions and photos of preserved shark and ray specimens. Then, students were given two and a half hours to identify 34 specimens to a predetermined taxonomic ranking: order, family, or species. During that period, the professor answered clarifying questions about dichotomous key terminology or difficult-to-identify specimens using the chat function in Zoom. Finally, at the end of the period, correct answers were shared and discussed with the class.

**Assessment Type:** Lab

**Activity Details:** First, refamiliarize yourself with the morphological characteristics of sharks and rays we learned in lecture 1. Some of these will be obvious to you by now (e.g. dorsal fin), while others will require some review (e.g. pectoral fin insertion). You can use your notes from the external morphology lecture as you work to identify these species, but the more familiar you are with these terms, the better.

Second, refamiliarize yourself with the 8 shark orders and 5 ray orders we discussed in lecture 3. Remember, each of these 13 orders are defined by a few unique characteristics. During this activity, some individuals will only need to be identified to order, while other individuals will need to be identified to species.

Third, download and examine the dichotomous key, and download the Google Slides file. Once you have these materials, you and your partner will have two and a half hours to identify the 34 individuals. Once you’re finished, we will review the correct answers.

When you have questions, please enter them into the “group chat” rather than the “individual chat,” and I’ll provide assistance. There’s a good chance that someone else can benefit from the answer to your question. Good luck, and be amazed at the diversity of sharks and rays in our region!

**Tips for executing this activity successfully online:** The key to this activity is making it a competition. The prize for the pair with the most correct answers can be small (e.g., a few additional points added to their lab grade), but the competitive aspect of the exercise creates an engaging atmosphere for the students. In addition, limit the number of specimens and when possible, use species that are found in the region. For this exercise, we provided standardized photos (e.g., we always used a lateral view and a dorsal view) of 34 sharks, skates, and rays found in the region where the class is taught. Make sure to allow sufficient time at the end of the exercise for review and reflection. Rather than simply providing the correct answers, elicit responses from the students and encourage them to share the answer they arrived at through the dichotomous key. This provides an opportunity to clarify confusing aspects of the key, and highlights the difficulty of distinguishing between similar species. Finally, make connections. Remind the students that without the mundane external morphology they learned at the beginning of the course, they would be unable to apply their knowledge to the dichotomous key. This exercise works best with groups of 30 students or less, and when grades are assigned based on completion (rather than the number of species correctly identified).

**Appendix B.3.** Biological hierarchy

**Learning objective category:** Application of Concepts/Hypotheses/Theories

**Course & Course Level:** Biology at the introductory undergraduate level. This activity could be modified for any course (e.g., introductory science courses, botany, ecology) where the learning objective is to apply knowledge of a hierarchical classification system.

**Class Type:** Lecture; On-line

**Class Size:** 100+

**Intended Audience:** Ecology-Related Sciences Major; Non-Ecology-Related Sciences Major; Non-Traditional Student; 2-year College; 4-year College; University

**Required Learning Time:** 30-minute portion of one 1.25-hour class period

**Prerequisite Student Knowledge:** Students should be familiar with the hierarchy of biological classification from atoms to the biosphere and abiotic and biotic components of ecosystems.

**Prerequisite Teacher Knowledge:** Instructors should be familiar with hierarchical classification, hierarchy of biological classification system, abiotic and biotic components of ecosystems, and first letter strategies (e.g., developing acrostics).

**Description:** This activity was developed as a lecture summary activity to aid students in applying the concepts and terminology associated with the hierarchy of biological organization (e.g., biosphere, ecosystems, communities, and so forth). Students applied knowledge by developing their own terminology definitions and study aids. Next, students applied definition of hierarchy by developing a hierarchical relationship of “everyday” objects (e.g., balls, writing utensils). Finally, students applied definitions of terms to classify levels within the biological hierarchy through critical thinking applications and drawing activities.

**Assessment Type:** Assignment (completed after lecture)

**Activity Details**

1. Define Hierarchy in your own terms.

2. Come up with a sentence, using the first letter of each group, to memorize the order for the Hierarchy of Biological Classification.

3. Based on the 5 objects below, develop your own 3-Level classification hierarchy to group the objects (in the worksheet: each of the 5 objects below are not listed, but rather images of each one are provided).

**Soccer Ball, Basketball, Pencil, Notebook, Pen**

Level I should contain all 5 objects, Level II should contain at least two groups of 2-3 objects, and Level III should contain at least three groups of 1-2 objects. For each level, list the main classifying characteristics for each group and draw/list which object(s) are in the group(s).

**Level I: (Hint: where can all of these objects be found?)**

**Level II:**

**Level III:**

4. Answer the following two questions based on the first hierarchical level you would encounter as you move from the most restrictive level (at the base of the hierarchy) to the most inclusive level (or most broad grouping level).

a. Based on the definition of Biology, which level of the Hierarchy of Biological Classification would first be classified as biotic?

b. Based on the definition of Organic, which level of the Hierarchy of Biological Classification would first be classified as biotic?

5. Draw the following (in the worksheet: each of the levels listed below are in a textbox with the level written in the top left corner);

**Ecosystem, Community, Population, Organism**

**Tips for executing this activity successfully online:** When developing worksheet-type active learning activities for students to apply knowledge from the lecture material, keep the activity (1) short (two pages works well) and (2) focused on the learning objective(s). Because you are not in the “classroom” with the student, make sure your directions are well written and supplemented with clarifying statements and hints. For example, in question three, I asked a “hint” question to help direct students’ reasoning. Additionally, in question four, I added alternative definitions in parentheses to help clarify the directions. Require that the activity be submitted. Provide students with an answer key so they can self-correct their answers. Although you are not correcting the activity, do provide feedback to the student. The feedback should highlight (1) which components were completed well and (2) which components should be further investigated before checking the answers. Before you submit feedback comments to the students, ask yourself these two questions: (1) does the feedback I provided encourage students to put forth effort when completing the activities? and (2) is the feedback constructive (i.e., does not discourage students from honestly answering questions)? A feedback comment that is specific to a student’s answer alerts the student that you did read their answers. In my experience, this often encourages students to put forth more effort when answering questions. When an answer is incorrect, softer language, such as, “Good start, but maybe take another look at question #X before you check your answers” alerts the student that you are reading his or her responses, does not discourage him or her from answering honestly, and encourages him or her to view the answer key and pay closer attention to, and learn from, the incorrect answer.

**Appendix B.4. Appendix B.4.** Coastal restoration plan

**Learning Objective Category:** Application of Concepts/Hypotheses/Theories

**Course & Course Level:** Coastal Restoration at the split upper undergraduate/graduate level. This activity could be modified for any course where a learning objective is to apply knowledge for the conservation and/or restoration of natural resources (e.g., wildlife management, fisheries management, natural resource management, conservation, etc.).

**Class Type:** Lecture/Lab; On-line

**Class Size:** 1-50 students (ideally < 30)

**Intended Audience:** Ecology-Related Sciences Major; Non-Traditional Student; 4-year College; University

**Required Learning Time:** One term (Maymester, summer term, full quarter, or semester)

**Prerequisite Student Knowledge:** Students should have a general understanding of ecology, biology, and some physical environmental processes.

**Prerequisite Teacher Knowledge:** Instructors should be familiar with biological/physical interactions, applied research, project planning, permitting, and budgeting for the general focus region of the course (e.g., coastal Mississippi, Southeastern USA, etc.).

**Description:** This two-part activity (1) was developed to help students critically think through the full process for restoration project development and immediately apply knowledge gained by creating a tangible product and (2) allowed for self-assessment of knowledge gained throughout the course by having students revise the product developed on day one into a final product turned in at the end of the course.

**Assessment Type:** Assessment of individual student performance; Create a diagram, drawing, figure, etc.; Give an oral presentation; peer evaluation

**Activity Details**

Restoration plan instructions - Please draft a short restoration plan for a habitat of your choice. Be as specific and as realistic as possible with the location of the target restoration area. Required plan elements are outlined below, but include the rationale/need, scope of work, anticipated benefits (outputs and outcomes), permitting considerations, monitoring, and budget (spreadsheet format). The total plan should be single spaced, use a maximum of 12 pt font, and be less than 5 pages in length (including figures and tables).

Restoration plan template

Project title: Provide a concise title that describes your project

Rationale/need: Describe why the project you are proposing is needed. Include sufficient background on the area so that reviewers can clearly discern the need for your project

Scope of work: List the overall goals of your project and describe how you will complete your project, including a timeline of activities

Anticipated benefits (outputs and outcomes): Describe the specific benefits you anticipate from this project, including both outputs and outcomes. This section will likely contain some elaborated text/concepts from the Rationale/need section

Permitting considerations: Describe any permits that might be needed to complete this work

Monitoring: Describe how you will determine if your project is successful at achieving the anticipated benefits described above

Budget: Provide an itemized budget for your project in spreadsheet format. This budget should be as detailed as possible and include, at a minimum, annual individual expenses within the categories of personnel, fringe benefits, travel, supplies, equipment, contractual, other, indirect costs, and total costs.

**Tips for executing this activity successfully online:** This activity was developed for, and is most effective in, relatively small class settings (i.e., < 30 students). The most important aspect of this activity is to allow the students to work through the process of developing a plan independently. They should go through the process of finding all their information independently and figure out what questions should be asked. To enable this, it’s important to maintain your role, not as the instructor, but as a potential funder, permitting agency, or stakeholder when discussing the students’ plans throughout the duration of the course. Expect playing these roles to account for a substantial amount of time and effort and, in some instances, considerably more time and effort than more structured activities. Help guide students to the information they need to answer most questions, rather than directly answering them. Another critical component of this activity is to allow time for students to discuss their project ideas and leverage the research of their classmates (i.e., independent group learning). In an online format that utilizes classroom management software (e.g., Canvas, Google Classroom, etc.), this can be done by setting up different threads/channels/message boards for students’ restoration plan questions directed to the instructor and to the rest of the class.

**Appendix B.5.** Restoring native prairie plant community

**Learning objective category:** Management of Natural Resources

**Course & Course Level:** Wildlife Plant Identification lecture course at a split upper undergraduate/graduate level. This activity could be modified for any course (e.g., ecology, wildlife management, natural resource management) where the learning objective is to evaluate a tool used to restore a native ecosystem.

**Class Type:** Lecture; Lab; Lecture/Lab; On-line

**Class Size:** 1–25

**Intended Audience:** Ecology-Related Sciences Major; Non-Traditional Student; 2-year College; 4-year College; University

**Required Learning Time:** Two 1.25-hour class periods

**Prerequisite Student Knowledge:** Students should be familiar with early seral stage plant communities, growth requirements of herbaceous plants, growth duration of plants, how to navigate the USDA Plants Database, and resources to utilize when conducting Internet searches for data.

**Prerequisite Teacher Knowledge:** Instructors should be familiar with plant community succession, growth requirements and growth duration of plants, native prairie plant community management and restoration, wildlife species and their use of early seral stage plant communities, local commercial seed mixes, USDA Plants Database, and Internet resources for data collection.

**Description:** This activity was developed as a lecture summary activity to provide students with skills to evaluate the effectiveness of a real seed mixture used for restoring a native prairie plant community as wildlife habitat. Students were given the common names of plant species as listed on an actual seed mix packet that was used to restore native prairie plant community in the Southeastern United States. Next, students were instructed to research background information (e.g., growth duration, native range, growing conditions) about, and wildlife use of, each plant species. Finally, students were asked to evaluate the effectiveness of this seed mixture and support their assessment with facts gathered from their research.

**Assessment Type:** Assignment (completed after lecture)

**Activity Details**

*Local Prairie Plant Community:*

As a wildlife or conservation professional, you may be tasked with evaluating a seed-mix to determine if it is appropriate for planting to restore native prairie habitat. Let’s practice with the seed mix label provided to us by Ken Knight, NC Supervising Wildlife Biologist with NC Wildlife Resources Commission.

Conduct Google searches for scientific names and utilize the USDA Plants Database and other reliable plant databases to evaluate the seed mix of forbs. Complete the table below (Figure B.5). Then, in a few sentences, summarize the “value” or “effectiveness” of the seed mix for native prairie habitat restoration in eastern USA.

| Scientific Name  (Common Name) | Annual or Perennial? | Native Range Description | Growing Condition Notes | Wildlife Use  (food, cover, both, part of plant, class Aves, Mammalia, both) |
| --- | --- | --- | --- | --- |
| (Lance-leaved Coreopsis) |  |  |  |  |
| (Plains Coreopsis) |  |  |  |  |
| (Black-eyed Susan) |  |  |  |  |
| (Bur-marigold) |  |  |  |  |
| (Goldenmane Tickseed) |  |  |  |  |
| (Narrow-leaved Sunflower) |  |  |  |  |
| (Spotted Beebalm) |  |  |  |  |

Figure B.5. Sample seed mix evaluation worksheet.

Describe the effectiveness of this as a native seed mix to restore prairie habitat in eastern USA. Use the ease of identifying the common-named species to scientific name, the growth duration, native range, growing conditions, and wildlife use to support your answer.

**Tips for executing this activity successfully online:** First, help students see the value in completing this activity; I took three specific steps to accomplish this task. First, I stated that this activity simulates a task the students may be required to complete in their future careers. Second, I required that the students watch a “Conversations with Professionals” video I recorded with Ken Knight (who is referenced in the activity). In the video, students (1) learn about the job tasks of a wildlife biologist with a state wildlife agency, (2) learn about the process Ken used to restore a field invaded by a non-native plant back to native prairie habitat, and (3) view the restored field. Third, I reiterated to the students that the seed mix they were evaluating is the one Ken used (i.e., it is an actual seed mix sold to the public, not just one I “made-up”). After completing these three steps, I provided the students with instructions for completing the task. For example, I supplied students with names of resources (e.g., Google search and USDA Plants Database) they should use during the activity. Because students are not in the “classroom” with you, it is important to offer guidance to help them formulate their answers to open-ended questions. For example, I provided a list of factors for students to consider when evaluating the effectiveness of the seed mixture. I gave students the answer key for this assignment if they received 100% participation points for completing the activity. Although you are not correcting the activity, do provide feedback to the student. The feedback should highlight (1) which components were completed well and (2) which components should be further investigated before checking the answers. Before you submit feedback comments to the students, ask yourself these two questions: (1) does the feedback I provided encourage students to put forth effort when completing the activities? and (2) is the feedback constructive (i.e., does not discourage students from honestly answering questions)? A feedback comment that is specific to a student’s answer alerts the student that you did read their answers. In my experience, this often encourages students to put forth more effort when answering questions. When an answer is incorrect, softer language, such as, “Good start, but maybe take another look at question #X before you check your answers” alerts the student that you are reading his or her responses, does not discourage him or her from answering honestly, and encourages him or her to view the answer key and pay closer attention to, and learn from, the incorrect answer.

**Appendix B.6.** Google Earth Mississippi estuaries journey

**Learning Objective Category:** Management of Natural Resources

**Course & Course Level:** AP Environmental Science (introductory level college equivalent), Honors Marine Biology (High School), Student Naturalist Program (Master Naturalist, Informal Education). The concept of this activity could be adapted globally to any region and the questions can be modified to suit a multitude of environmental, geographic, and anthropological courses where the learning objective is management of natural resources.

**Class Type:** Lab; Lecture/Lab; Seminar; Discussion Section; On-line

**Class Size:** 1-25, 12 or less is ideal

**Intended Audience:** Ecology-Related Sciences Major, Non-Traditional Student; 2-year College; 4-year College; University; Informal Education, High School

**Required Learning Time:** One 1-hour class period, can be extended depending on depth of discussion and time given to plan solutions

**Prerequisite Student Knowledge:** Students should have knowledge of basic coastal ecology and a conceptual understanding of ecological impacts from anthropogenic activity.

**Prerequisite Teacher Knowledge:** Instructors should have knowledge of local geography, coastal ecology, and the effects of anthropogenic activity.

**Description:** This activity encourages students to discuss management of natural resources through a tour of local regions that are impacted by anthropogenic activity. First, students enter GPS coordinates into the Google Earth program; these initial coordinates virtually place them upriver in the Pascagoula River. They are then instructed to move in predetermined directions throughout waterway in the region, stopping at specific areas to try to determine what activities (e.g., golf courses, mining, refineries, roadways) they might see that are impacting that area as well as adjacent habitat. Lastly, the students are asked to supply and discuss potential solutions for reducing such impacts.

**Assessment Type:** If completed in synchronous setting with instructor present - Assessment of student groups/teams/individuals through discussion
If completed in a non-synchronous setting - Answer short answer questions

**Activity Details**
We are going to take a virtual journey through the Pascagoula River and Grand Bay estuaries using Google Earth. You can use Google Earth at<https://www.google.com/earth/> by opening the page in a Google chrome browser or by downloading the desktop version of the application. Throughout this trip you will apply your knowledge of geological formations within the estuaries as well as how humans alter ecosystems to explain potential impacts that you see along the journey as well as offer solutions to mitigate those impacts.

**We will begin our journey upriver on the Pascagoula River. Enter the following into your Google Earth search bar: 30.600734 -88.631144.**

1. Zoom out to a higher altitude. What do you notice about the path the river flows at this point? Is it straight? Explain why the river does this?

2. Zoom back in. What do the riverbanks in the immediate vicinity look like? Are they natural or man-made? What does this tell you about this area?

3. Let’s start moving down the river. As we pass by some of the houses and docks, you’ll notice some sand bars on the insides of the riverbank bends. How do they form? How might they be affected by boats from these docks parking along the sand bars or by boats moving through these waters at high speeds? What sort of impacts might be felt further downstream from this? How can this be prevented?

4. Keep heading down the river. You will come across a major industrial site with some very dark colored pools/ponds. What might they be, how could they impact the waterways if there were flooding, and what can be done to reduce the chance of that?

5. When you reach a fork in the river, head west. Follow it south until you reach a large populated area along the riverbank. What development do you see that could be a source of a large quantity of nitrogen and phosphorus entering the estuary? What might be the result of this? How can it be mitigated?

6. Let’s head back north to the fork in the river. This time we’ll take the east branch. After you cross highway I-10, look southeast and you will see a road that crosses over the mouth of the Escatawpa River Marsh Coastal Preserve. Notice anything interesting on that road? Depending on exactly what the composition of those mounds are, they could contain minerals such as calcium carbonate or iron sulfate. How could they impact our waters and how can that be prevented?

7. Follow the river south through the shipping canal and port. What do you notice about the shape of the river through this area? What about the riverbanks? Why do you think this has occurred and what effects might it potentially have? How do the pros and cons of this type of shoreline compare to a living shoreline?

8. Just south of the port, in the Mississippi Sound, you will see an island with a naval base. This is Singing River Island. How do you think it formed? What changes to hydrology and sediment transport could that process bring about?

9. Keep heading south. You will see some long skinny islands that stretch from east to west. What type of islands are they and how do they impact the estuary?

10. Zoom out so you can see the entirety of the two barrier islands south of the river, Horn Island and Petit Bois Island. On the top of the screen, travel back in time by looking at historical imagery of the barrier islands. What do you notice about them that might tell you how they form? To the east is another barrier island, Dauphin Island. This island is governed by the same processes, but it is developed and populated by humans. Why might that be an issue when it comes to such processes? What can be done about it?

11. We are going to head over to Grand Bay to the northeast, but first we will head directly north of Petit Bois Island to look at two industrial sites. What do you see and how might they impact the estuary?

12. Although there may be some negative impacts from these industries, how can these industries benefit the people in this region? Assess the pros and cons of the impact of these industries versus the benefits they provide.

13. Head northeast into Grand Bay NERR. Zoom out until you can see all the way north to Highway 90 as well as west to the Pascagoula River. Compare the width of the rivers feeding Grand Bay estuary with the Pascagoula River Estuary. How do you think that affects the water conditions in the two estuaries?

14. Do you notice anything that could potentially alter the flow of the Escatawpa River? How might that impact Grand Bay? How could it be restored?

15. If you could compare the organisms and their locations between the two estuaries, what similarities and differences do you think you would see?

16. Human impacts such as runoff of nutrients or habitat loss can occur on large scales such as within the estuary, but can also occur and be contributed to on small, individual levels. What can you do personally to reduce impacts on our waterways?

Note: This activity was inspired by a lesson titled “A Trip Down the Alabama River” in the Estuaries 101 curriculum from the National Estuarine Research Reserve System and the National Oceanic and Atmospheric Administration. <https://coast.noaa.gov/estuaries/curriculum/>

**Tips for executing this activity successfully online:** This activity works best in a synchronous setting whereby the instructor shares his or her screen with the class. This allows for group debate and discussion at each stopping point. The activity can work well in small classes (< 10 students) with students working individually, or in large classes with students working in teams. If the students are working in teams and the technology is available, then the instructor should allow the teams to break off into individual discussion rooms. This increases student-to-student communication through group development of ideas. Moreover, the activity can be modified into a game that rewards the team who presents the best mitigation strategies. This activity should be student-driven, with the instructor functioning mostly as a facilitator. Specifically, the instructor should act as a “tour guide” by navigating the virtual map from location to location and only provide guidance if the students seem to be unable to identify an important aspect of the question. A key aspect of this activity’s success is the written description, provided on the student worksheet, for each stop on the virtual map tour. These details are especially important if the instructor records the virtual tour and provides it to students who were unable to join the synchronous class session.

**Appendix B.7.** Initial and reply discussion board posts

**Learning objective category:** Development of Professional Skills

**Course & Course Level:** Environmental Law at the introductory undergraduate level. This activity could be modified for any course (e.g., introductory or upper undergraduate or graduate levels) where the learning objective is to develop professional opinions and practice communication skills.

**Class Type:** Lecture; Seminar; Discussion Section; On-line

**Class Size:** 1–20

**Intended Audience:** Ecology-Related Sciences Major; Non-Ecology-Related Sciences Major; Non-Traditional Student; 2-year College; 4-year College; University

**Required Learning Time:** Two 1.25-hour class periods

**Prerequisite Student Knowledge:** Students should be familiar with the Administrative Procedure Act and how it applies to environmental laws and use of discussion board posts/threads.

**Prerequisite Teacher Knowledge:** Instructors should be familiar with the Administrative Procedure Act, federal and local environmental laws, local public comment forums related to environmental laws, and discussion board forums in learning management system (e.g., Canvas, Blackboard).

**Description:** This activity was developed as a lecture summary activity to provide students with the opportunity to apply environmental laws to their professional life and practice communication skills. Students were provided web links to administrative code posted in a state town hall forum as required by public notice executive orders. Next, students were asked to write an initial discussion board post in which they formulated their opinion on three sections of the Administrative Code that were listed; two Administrative Code sections were chosen by the instructor and one was chosen by each student. Two days after the initial post was due, students were required to post a reply to any one classmate’s initial post. For the reply post, students were asked to explain if they agreed or disagreed with their classmate and why.

**Assessment Type:** Assignment (completed after lecture)

**Activity Details**

**Discussion Board: Public Notice**

You learned that part of the administrative procedure process (APA) involves the public. This applies to Virginia; see VA Executive Order [http://dpb.virginia.gov/regs/EO17.pdf](about:blank). The state of Virginia has an on-line public comment forum on Virginia Regulatory Town Hall ([www.townhall.virginia.gov](about:blank)).

**Initial Post - Due Date #1**

- Click on the link below or copy and paste the link and read the sections listed below regarding VA administrative code 19VAC15-20.
- Make sure the link takes you to: Administrative Code: Table of Contents » Title 19. Public Safety » Agency 15. Department of Fire Programs » Chapter 20. Regulations Establishing Certification Standards for Fire Inspectors »
- [https://law.lis.virginia.gov/admincode/title19/agency15/chapter20/](about:blank)
- **Read Section 10 (Definitions) through Section 190 (General)**
  - Each section is fairly short (Section 10 is longest)
  - You can easily navigate from one section to another by clicking on the section arrow button located at the top and bottom of each web page.
- This regulation is up for review. Click on the below link to view the post on the Virginia Regulatory Town Hall or if link no longer exists, then see post in PDF file named, “DB#2B_VirginaTownHallPost_19VAC15_20“
  - [http://townhall.virginia.gov/L/comments.cfm?periodicreviewid=1616](about:blank)
- **While the public comment period on the Virginia Regulatory Town Hall forum ended at 11:59pm on 1/25/2018, you will enter your comments and suggestions as your initial discussion board post regarding the following parts of this regulation;**
  - Section 80: What is your opinion regarding the minimum number of hours required for training with experienced personnel written in the regulation?
  - Section 130: What is your opinion regarding the minimum number of questions required to be asked to evaluate a student's understanding of instructional material?
  - Section ?: You pick another item to comment on; be sure to reference the section it is found under so other classmates can easily find it.

**Reply Post - Due Date #2 (2-3 Days after Initial Post Due Date)**

- Choose to reply to at least one initial post from your fellow classmates.
- Do you agree? Disagree? Why?
- Remember to be courteous to maintain a healthy debate. Everyone is entitled to their opinion, so I’ve been told.

**Tips for executing this activity successfully online:** The most important aspect for successful completion of this activity are the two separate due dates. The online instructor must facilitate the assignment of tasks so they can be completed successfully. In my experience, most online students will wait until the due date to turn in an assignment or complete a discussion board post. The two separate due dates facilitates communication between students by requiring all students to post their initial “thoughts'' by the first due date, thereby allowing students ample time to review the initial thoughts and formulate a response by the second due date. For this particular activity, two days was plenty of time for students to review posts and reply in a class size of ten students or less. Additional time to review initial posts may be required if the class size is larger. The online instructor should also make the activity applicable to the course and the lives of the students. For example, I reminded students that they already learned about the public notice requirement in the lecture material. Additionally, because the course textbook discussed federal laws, I showed students how it was similar to state laws. I selected to use the state of Virginia because most of my students lived and worked in Virginia. If the online instructor chooses to use web links, then he or she should check the web links before the activity is due to ensure they are working properly, and also write a description of the web link. It is a good idea to inform students that if a web link is broken, they should notify the instructor and complete a Google search to attempt to find the redirected web link. It is just as important to provide guidance as it is to provide student choice. For example, I selected a couple of administrative codes for students to comment on, but I also allowed students to express their opinion about a code that they found interesting. The online instructor should always remind students about discussion board etiquette. Even though I assigned several two-reply discussion board tasks throughout the course, I always posted the same phrase at the end of every assignment description: “Remember to be courteous to maintain a healthy debate. Everyone is entitled to their opinion, so I’ve been told.” I like to show my humor in my written directions. I think it helps my students “see” my personality through my written communication with them. Lastly, do not comment in a discussion board forum intended to increase student-to-student communication. However, I did communicate with each student when I provided feedback as I graded their posts on the merits of completion and effort.

**Appendix B.8.** Field notebook

**Learning Objective Category:** Development of Professional Skills

**Course & Course Level:** Field Ornithology/Coastal Birds of Alabama at the introductory and upper undergraduate level. This exercise could be modified for any field-based course that emphasizes development of the descriptive and documentary record-keeping skills essential to professional field research and monitoring (e.g., general ecology, field botany, marine ecology, wildlife management, herpetology, etc.)

**Class Type:** Lecture/Lab (field); In-person and On-line

**Class Size:** 1-20 (ideally <15)

**Intended Audience:** Ecology-Related Sciences Major; Non-Ecology-Related Sciences Major; 4-year College; University

**Required Learning Time:** One term (Maymester, summer term, full quarter or semester; on-going activity during field sessions requiring 5-20 mins/hr of field time

**Prerequisite Student Knowledge:** No specific prior knowledge required; this is a formative exercise.

**Prerequisite Teacher Knowledge:** Instructors should be familiar with basic standards for annotation, description, taxonomic nomenclature, and meta-data for field observations and data collection within their discipline.

**Description:** Creating and maintaining a field notebook supports the development of multiple, broadly transferrable professional skills, including (1) accurate record keeping, (2) information management, (3) data collection, (4) observation and description, (5) connecting direct experience with broader theories, models, and hypotheses, (6) identifying questions, and (7) self-directed inquiry. Students are required to keep detailed field notebooks to record field observations and standardized data in real time using a model format based on exemplary styles (e.g., Remsen, 1977; Herman, 1986; Montgomerie, 2018). Students submit notes periodically during the course to receive feedback, coaching for improvement, and prompts to direct future observations. Field notebooks are recognized as a high-impact learning activity and are commonly assigned in field-based courses within the natural sciences (Farnsworth et al., 2014).

**Assessment Type:** Assessment of individual student performance

**Activity Details**

Initial Field Notebook instructions -- You are expected to spend approximately 6-10 hrs/week engaged in independent field study. You will document your observations, experiences, skill development, data, and personal goals in a field notebook.

Your detailed field notebook will include all basic information relating to daily birding activities (e.g., location, time, weather conditions, species list with the number of individuals observed) and specific observations (e.g., behavior, habitat characteristics, other organisms observed) recorded as lists, data, narrative, and sketches as appropriate. On some occasions, you will collect specific data related to behavioral ecology, diversity, populations, etc. Be sure to include detailed descriptions of methodology in your notebook such that someone could pick up your notebook and repeat your exact methodology along with data or summaries as appropriate (sometimes data will be collected on separate data sheets). The instructor will work with each of you individually to improve your note-taking efforts. The very best field notebooks will include reflections on skill development, questions, and/or hypotheses.

Your field notebook will follow a standard format derived from the work of Joseph Grinnell (Herman 1986) and J. Van Remsen, Jr. Please review “On Taking Field Notes” (Remsen 1977) and “Joe Grinnell’s Notes” (Montgomerie 2018) and examples provided by the instructor. Use the accompanying template to guide you in keeping key information organized and maintaining consistency.


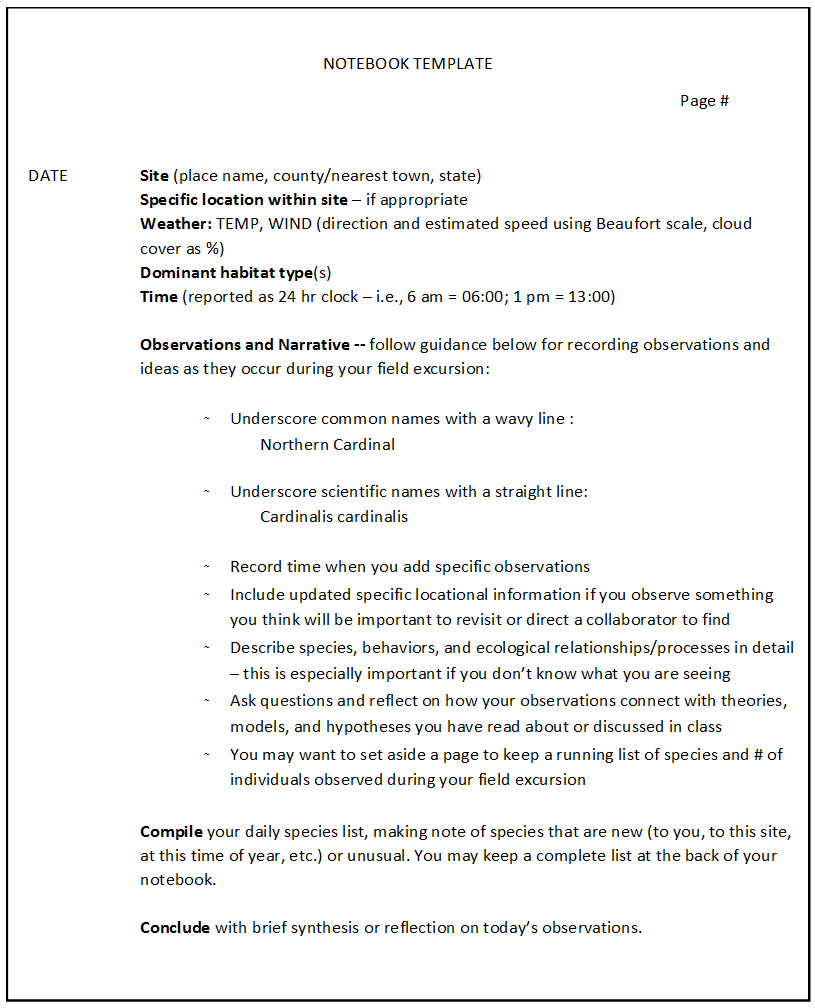


Figure B.8. Example field notebook template including critical data fields and types of observation elements.

**Tips for executing this activity successfully online:** This assignment was designed for extended class field excursions and successfully adapted to online instruction and independent field study. Prior to students’ first field excursion, provide them with examples of completed field notebooks and discuss the elements of the template during synchronous class meeting or virtual office hours. In addition, help guide students with respect to organizing their field notebooks during synchronous class sessions or through a short (3-5 minutes) pre-recorded video. This should include a discussion the field notebook criteria, which will improve the student’s understanding of, and purpose behind, each field notebook criterion (see Farnsworth et al. 2014, pp. 14-15, 18-20). Provide students with a detailed assessment rubric of the field notebook criteria that will be used to evaluate their field notebooks. The notebook can also be used to assess participation and engagement. It is important that students are required to turn in samples from their notebook at regular intervals. This ensures students are keeping up with field study and note-taking and provides an opportunity for specific feedback. Instructor feedback should focus on encouragement and further prompts for observation, description, and data collection. Feedback can help students to develop the ability to record descriptive detail and frame and/or contextualize their observations within ecological theory, principles, and/or hypotheses. Assigning linked projects including essays, literature reviews, and data analysis that build on direct observations, data, and reflections recorded in the notebook will help students see the value of note-taking. Encourage students to purchase a bound field notebook with water-resistant paper (e.g., *Rite in the Rain* bound notebooks) and remind them to use pencil or waterproof pens to avoid “bleeding” and blotching and ultimately the loss of valuable field observations.

**Appendix B.9.** Peer evaluation

**Learning objective category:** Evaluation of Concepts/Practices

**Course & Course Level:** Aquatic Biodiversity Conservation at the graduate level. This activity could be modified for any course (e.g., introductory, advanced undergraduate, graduate) where the learning objective is for students to develop original works and practice their evaluation skills.

**Class Type:** Lecture; On-line

**Class Size:** 1–10

**Intended Audience:** Ecology-Related Sciences Major; Non-Traditional Student; 4-year College; University

**Required Learning Time:** 4-weeks or eight 1.25-hour class periods

**Prerequisite Student Knowledge:** Students should be familiar with freshwater streams and rivers; ecology, taxonomy, biodiversity, conservation and management, basic design of interpretive educational displays for the public, and sound evaluation practices.

**Prerequisite Teacher Knowledge:** Instructors should be familiar with freshwater streams and rivers; ecology, taxonomy, biodiversity, conservation and management, interpretive educational displays design, evaluation methods, development of rubrics, and assigning peer evaluation assignments in learning management system (e.g., Canvas, Blackboard).

**Description:** This activity was developed as a lecture series summary and exam review activity to provide students with an opportunity to practice evaluation of peers’ conservation education interpretative displays. Students were provided with resources, examples, and rubrics to develop their own interpretive display on a topic that will be selected by each student, but related to material from the lecture series on freshwater river ecosystems. Next, students are given instructions regarding evaluation. Finally, students use a faculty-developed grading rubric to evaluate classmates’ interpretive displays on the merits of creativity, alignment of learning objectives, scientific accuracy, and execution.

**Assessment Type:** Assignment (completed after an exam period lecture series)

**Activity Details**

Freshwater Streams & River Interpretive Display

You will design an interpretive display on a topic of your choosing related to freshwater streams and rivers. The conservation education interpretive display must be based on scientific facts, but delivered in a manner easily understood by the general public. See Figure B.9 for an example Interpretive Display.

You will design the display in Microsoft PowerPoint. Reduce image file sizes to speed up your assignment upload. Then, you will save the file as a PDF file before uploading the assignment.

Make sure to re-review the previous section on tips to follow when designing interpretive displays. Additionally, review the grading rubrics (completeness and peer-evaluation). I will evaluate your work on the merits of completeness (includes the entire bulleted list below). Your classmates will evaluate your work on the merits of creativity, alignment of learning objectives, scientific accuracy, and execution.

Do not include your name or identifying features in your submitted assignments. These will be reviewed by your peers.

Completeness (Part 1 of 2): Worth 80-points of total, 140-points (each section below is worth 20-points)

- Topic: Single subject related to freshwater stream and river ecology & 3 main learning objectives (3 main sections of the display)
- Include 2-3 high quality photos that helps create a connection and supplements the text
- Text is ~250 words; in total ~15-17 sentences. For example, title, headings and 3-5 sentences per learning objectives section.
- Screen shot of Readability Statistics of your text in Microsoft Word on separate slide. Reading ease should be at least 60 & grade level should be between 6.0 to 8.0 (corresponds to 6-8th grade reading level).

The peer review assignment will be completed next week. Here is an overview of the criteria your work will be evaluated on.

Peer-Review (Part 2 of 2): Worth 60-points of total 140-points (each section below is worth 10-points). TIP- review as if this was your own paper. Be honest. If the criteria described in the rubric is met, then award those points (Table B.9). No partial point assignments are allowed.

- Title: title draws the reader in and is less than 7 words
- Learning Objective 1/Section 1: related to the main theme and the entire section is focused on the main learning objective subject.
- Learning Objective 2/Section 2: related to the main theme and the entire section is focused on the main learning objective subject.
- Learning Objective 3/Section 3: related to the main theme and the entire section is focused on the main learning objective subject.
- Accuracy: the information in the display is based on scientific facts
- At least 3 resources cited (in display or on a separate page with readability screenshot) from credible sources (textbooks, research manuscripts, websites from; higher educational institute (.edu), government agency (.gov), or conservation organization with specific knowledge of species such as a zoo or aquarium.
- Organization and execution: neatly organized, easy to read, good contrasting colors, sans serif font, high quality images.

Table B.9. Rubric for peer evaluation that will be used by each student to evaluate their peers’ interpretive displays. The rubric is provided to students with the assignment description.

| **Criteria** | **Ratings** | | | **Total Possible Points** |
| --- | --- | --- | --- | --- |
| **Title**  Draws reader in, is creative, is within 7 word limit, summarizes entire purpose of the interpretive display | **10.0 pts**  **90-100%** **Complete**  Title is catchy and summarizes the purpose of the display, is within the 7 word limit (allowed 1 extra word). | **5.0 pts**  **50-89% Complete**  Title does not describe the purpose of the display, is not creative, is more than 2 words over the 7 word limit | **0.0 pts**  **0-49% Complete**  Title is missing or no effort was put into title creation; ‘one-worder’ or more than 3 words over the 7 word limit | 10.0 pts |
| **Learning Objective 1**  Section 1 is related to the main theme (title). The section title clearly describes the learning objective, 3-5 sentences that are related to the learning objective | **10.0 pts**  **90-100% Complete**  The section title clearly describes the learning objective, 1 sentences is not related to the learning objective, within the 3-5 sentence limit (allowed 1 sentence forgiveness). | **5.0 pts**  **50-89% Complete**  The section title does not clearly describe the learning objective, 2-3 sentences are not related to the learning objective, not within sentence limit- less than 2 sentences or more than 6 sentences. | **0.0 pts**  **0-49% Complete**  Section title is missing or does not clearly describe the learning objective, more than 4 sentences are not related to the learning objective, not within sentence limit- less than 2 sentences or more than 6 sentences. | 10.0 pts |
| **Learning Objective 2**  Section 2 is related to the main theme (title). The section title clearly describes the learning objective, 3-5 sentences that are related to the learning objective | **10.0 pts**  **90-100% Complete**  The section title clearly describes the learning objective, 1 sentences is not related to the learning objective, within the 3-5 sentence limit (allowed 1 sentence forgiveness). | **5.0 pts**  **50-89% Complete**  The section title does not clearly describe the learning objective, 2-3 sentences are not related to the learning objective, not within sentence limit- less than 2 sentences or more than 6 sentences. | **0.0 pts**  **0-49% Complete**  Section title is missing or does not clearly describe the learning objective, more than 4 sentences are not related to the learning objective, not within sentence limit- less than 2 sentences or more than 6 sentences. | 10.0 pts |
| **Learning Objective 3**  Section 3 is related to the main theme (title). The section title clearly describes the learning objective, 3-5 sentences that are related to the learning objective | **10.0 pts**  **90-100% Complete**  The section title clearly describes the learning objective, 1 sentences is not related to the learning objective, within the 3-5 sentence limit (allowed 1 sentence forgiveness). | **5.0 pts**  **50-89% Complete**  The section title does not clearly describe the learning objective, 2-3 sentences are not related to the learning objective, not within sentence limit- less than 2 sentences or more than 6 sentences. | **0.0 pts**  **0-49% Complete**  Section title is missing or does not clearly describe the learning objective, more than 4 sentences are not related to the learning objective, not within sentence limit- less than 2 sentences or more than 6 sentences. | 10.0 pts |
| **Scientific Accuracy**  All facts on display are based on scientific facts. Cite at least 3 credible resources either in a dedicated section in the display or on a separate page with the readability screenshot. Credible sources include textbooks, research manuscripts, websites from; higher educational institutes (.edu), government agencies (.gov), or conservation organizations with species specific knowledge, such as a zoo or aquarium | **10.0 pts**  **90-100% Complete**  Cited at least 3 resources. 90-100% of resources are from credible sources. | **5.0 pts**  **50-89% Complete**  Cited only 2 resources and/or only 50-89% of resources are from credible sources. | **0.0 pts**  **0-49% Complete**  Cited only 1 resource and/or only 0-49% of resources are from credible sources. | 10.0 pts |
| **Organization and Execution**  Neatly organized, easy to read or good flow, good contrasting colors, sans serif font, font size is large enough to make it easy to read, creative, adhered to given ADA standards. | **10.0 pts**  **90-100% Complete**  6-7 of given criteria were followed. | **5.0 pts**  **50-89% Complete**  3-5 of given criteria were followed. | **0.0 pts**  **0-49% Complete**  0-2 of given criteria were followed. | 10.0 pts |
| **Total Points:** | | | | **60.0 pts** |


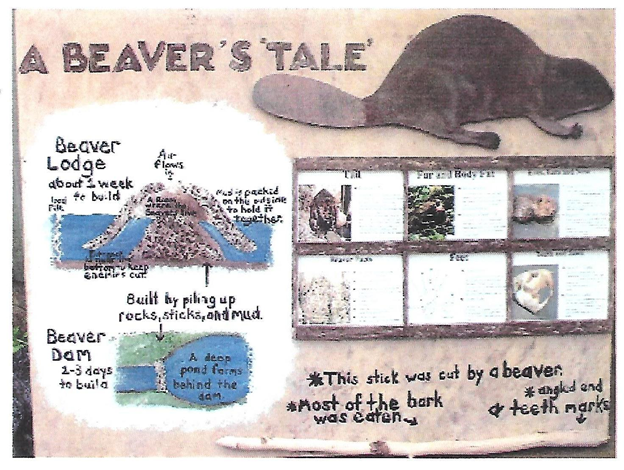
Figure B.9. Example of an interpretive display I developed while working as a NC State Park Ranger in 2003. Examples are important to provide so students will have a better understanding of how to design an original product. Because the image of my interpretive display is lower quality which makes it hard to read all of the text, I provided students with a description of the important aspects of the display, “Key Components: (1) Catchy title focused on a single subject matter: Beavers; (2) Three main sections that align with the learning objectives: a) Beaver Habitat (Far left section), b) Beaver Anatomy (Right, top section), and c ) Signs of Beaver Activity (Bottom, right section).”

**Tips for executing this activity successfully online:** While I have not personally conducted a peer evaluation assignment online, I have completed peer-to-peer evaluation work as an online student and have discussed peer evaluation/group assignments with other online students. The biggest complaint was that either other students waited until the last minute to provide their work or other students did not turn in their work at all to be evaluated. The online instructor must facilitate the peer evaluation for successful completion of the active learning activity. First, employ two separate due dates – one for turning in the assignment and another for completion of the peer evaluation. Second, assign the peer evaluation tasks to each student to complete. Next, provide students with guidance regarding how to conduct a peer evaluation. For example, I suggest providing students with a detailed rubric and instructed students that no partial points would be awarded. I also suggest reminding students that it is okay, when evaluating, to award all of the points if all the criteria outlined in the rubric were met. I suggest asking each student to complete 2-3 peer evaluations. In doing so, each student’s peer evaluation grade would be the average point value awarded from several peer evaluators. Lastly, student-to-student communication is important, but so is student-to-instructor communication. Evaluate the students’ work on the merit of completeness and provide each student with constructive feedback.

**Appendix B.10.** Evaluate results of published literature

**Learning objective category:** Evaluation of Concepts/Practices

**Course & Course Level:** Wildlife Plant Identification at a split upper undergraduate/graduate level. This activity could be modified for any course (e.g., ecology, zoology, wildlife biology) where the learning objective is to evaluate the results of published literature to determine if it supports a concept or practice.

**Class Type:** Lecture; Lecture/Lab; Seminar; Discussion Section; On-line

**Class Size:** 1–20

**Intended Audience:** Ecology-Related Sciences Major; Non-Traditional Student; 2-year College; 4-year College; University

**Required Learning Time:** Two 1.25-hour class periods

**Prerequisite Student Knowledge:** Students should be familiar with scientific methods and scientific studies (e.g., control and experimental units), early seral stage plant communities, plant community succession theories, disturbance impacts on plant community succession, and Aldo Leopold’s theory of the cow as a habitat management tool.

**Prerequisite Teacher Knowledge:** Instructors should be familiar with experimental design, plant communities and management, plant community succession theories, natural and human induced disturbances and their impacts on plant community succession, Aldo Leopold’s theory of the cow as a habitat management tool, and current published literature.

**Description:** This activity guides students through evaluating the results of published literature to determine if it supports a theory that has led to a common management practice. First, students are oriented to the learning objective for the activity in the assignment instructions: “Evaluate the results of the study to determine if it supports or does not support Aldo Leopold’s theory of using livestock as a wildlife habitat management tool; to set back the seral stage of succession by consuming grasses, thus increase abundance of forbs” (Leopold, 1933). Next, students are asked several key questions to guide them in pulling pertinent facts from the manuscript that provide supporting evidence for their evaluation. Finally, students are asked an open-ended evaluation question: “Did the researchers of this manuscript find a treatment effect that would support Aldo Leopold’s theory of using cattle or livestock as a wildlife habitat management tool to set back the seral stage of succession?”

**Assessment Type:** Assignment (completed after lecture)

**Activity Details**

**The Cow (Herbivores in Grasslands):** Read the following manuscript regarding the impacts of grazing by different large herbivores in grasslands and answer the questions. Evaluate the results of the study to determine if it supports or does not support Aldo Leopold’s theory of using livestock as a wildlife habitat management tool; to set back the seral stage of succession by consuming grasses, thus increase abundance of forbs.

**Read the following Manuscript:** Liu et al. (2015). Impacts of grazing by different large herbivores in grassland depend on plant species diversity. Journal of Applied Ecology 52: 1053–1062. Retrieve from: [https://besjournals.onlinelibrary.wiley.com/doi/epdf/10.1111/1365-2664.12456](about:blank)

**Answer the following Questions:**

1. What are the three main factors herbivores strongly influence in plant communities and ecosystem processes?

2. What type of plant community and climatic condition prevailed on the study site?

3. What herbivores were used in the study?

4. What is the purpose of the control sites in the low and high plant species diversity study sites? (For a visual aid, reference Figure 1)

5. Summarize the results by grazing treatment for;

a. Plant Diversity (Species richness):

b. Plant Cover:

c. Above Ground Biomass:

d. Plant Species and Function Group Composition (Grasses and Forbs):

e. Diet Overlap:

6. Did the researchers of this manuscript find a treatment effect that would support Aldo Leopold’s theory of using cattle or livestock as a wildlife habitat management tool to set back the seral stage of succession? Explain your answer.

**Tips for executing this activity successfully online:** First, in the instructions, remind students how the activity aligns with their lecture material and provide them with the final evaluation question (e.g., the learning objective). I briefly restated a summary of their lecture material on using cattle as a wildlife habitat management tool in grassland plant communities. The learning objective for this activity is written in the activity’s instructions so that the students will read the manuscript with this objective in mind. Second, guide students through the process for collecting key information that will help support their evaluation. I asked students about pertinent information from the introduction and methods, such as factors regarding plant communities, research area, and study design (i.e., livestock species utilized). Next, I reminded students to reflect on the purpose of a control by asking them about the control used in this study. Then, I guided students through summarizing the results by asking them to summarize the results of each section of the manuscript’s results. This helped students focus on one results section at a time. Finally, I asked the opened-ended evaluation question. We completed several of these types of activities throughout this course. For every activity, I asked 1-2 key, guiding questions from the introduction and methods sections. I also guided students through summarizing results by asking for the summary by sections listed in the manuscript or by providing hints for students to focus on. Then, I asked the final open-ended evaluation question, which echoed the learning objective listed in the instructions. I evaluated this activity on the merit of effort and completion and awarded participation points. If students received 100% participation points, then they were given access to the answer key to self-correct their answers. As I reviewed their answers, I provided constructive feedback to each student. I also included some additional guiding questions when students did not quite support their answer to help students focus on where they could have retrieved the supporting evidence for their evaluation. The feedback should highlight (1) which components were completed well and (2) which components should be further investigated before checking the answers. Before you submit feedback comments to the students, ask yourself these two questions: (1) does the feedback I provided encourage students to put forth effort when completing the activities? and (2) is the feedback constructive (i.e., does not discourage students from honestly answering questions)? A feedback comment that is specific to a student’s answer alerts the student that you did read their answers. In my experience, this often encourages students to put forth more effort when answering questions. When an answer is incorrect, softer language, such as, “Good start, but maybe take another look at question #X before you check your answers” alerts the student that you are reading his or her responses, does not discourage him or her from answering honestly, and encourages him or her to view the answer key and pay closer attention to, and learn from, the incorrect answer.
